# How Extreme Is It Anyways?: An Empirical Investigation Into the Prevalence and Strength of Extreme Response Style

**DOI:** 10.1177/00131644261435119

**Published:** 2026-04-09

**Authors:** Martijn Schoenmakers, Jesper Tijmstra, Jeroen Kornelis Vermunt, Maria Bolsinova

**Affiliations:** 1 Tilburg University, The Netherlands; 2 Netherlands Interdisciplinary Demographic Institute (NIDI), KNAW/University of Groningen, The Netherlands

**Keywords:** item response theory, extreme response style, generalized partial credit model, multidimensional nominal response model, IRTree model

## Abstract

Extreme response style (ERS), the tendency of participants to endorse the extreme categories of an item partially independent of item content, has repeatedly been found to decrease the validity of Likert-type scale results. For this reason, many IRT models have been developed that attempt to detect and correct for ERS. Despite the substantive literature on ERS and modeling of ERS, several important questions remain. To date, there is no clear estimate of how often ERS occurs in practice across a variety of scales and populations. In addition, there is little guidance on what item parameters for ERS models are commonly found in empirical data, while this information is crucial to inform future methodological studies utilizing ERS models. Finally, there is only limited information available on which ERS models tend to fit the data best. The current study sets out to address these three issues by analyzing data from the Programme for International Student Assessment using a generalized partial credit model, several multidimensional nominal response models, and several IRTree models. Results indicate an extremely high prevalence of ERS across scales, populations, and timepoints. Item parameters for future methodological studies are presented, and a general preference for IRTree models over MNRM models is found in many datasets. Implications for futures studies are discussed, and recommendations for practice are made.

Likert-type scales are widely used in the social sciences to measure a wide array of different latent traits (Nemoto & Beglar, 2014; Van Vaerenbergh & Thomas, 2013). The validity of utilizing Likert-type scale measurements can be compromised by response styles, the systematic tendencies for participants to respond in particular ways (partially) independent of item content (Falk & Cai, 2016; Van Vaerenbergh & Thomas, 2013). One of the most studied and encountered response styles is the extreme response style (ERS). ERS is defined as the tendency of respondents to choose the extreme options of a scale, partially independent of the actual question’s content (Greenleaf, 1992; Van Vaerenbergh & Thomas, 2013).

The presence of ERS may threaten the validity of Likert-type scales in several ways. First, ERS can distort group comparisons by biasing estimates of latent trait means and variances at the group level (Schoenmakers et al., 2024). Second, ERS may introduce construct-irrelevant variance into data, which can reduce the size of estimated effect sizes (Van Vaerenbergh & Thomas, 2013). As an example, failing to adjust for ERS in one study led to a drop in explained variance from 69.5% to 53.5% (Lau, 2007). Therefore, detecting and correcting for ERS is essential to ensure accurate measurement and valid conclusions (Schoenmakers et al., 2026).

While the effects of ERS on measurement are relatively well-known, less is known about the general prevalence and strength of ERS in empirical data across different populations and scales. Note that while this paper discusses the “prevalence” and “strength” of ERS, strictly speaking, ERS would only be detected if individuals differ in their ERS value, since no neutral ERS point exists naturally (see e.g., Bolt & Meng, 2025). The prevalence of ERS is thus used as a shorthand for the prevalence of individual differences in ERS throughout the paper, and the strength of ERS is used as a shorthand for the degree of variability between individuals in their tendency to choose extreme categories. While many studies have provided some information on the prevalence and strength of ERS, current investigations into ERS tend to limit themselves to either a single scale administered across several populations (e.g., Clarke, 2001; De Jong et al., 2008; Peterson et al., 2014; Schoenmakers et al., 2025) or several scales administered in only a single population (e.g., Naemi et al., 2009; Wetzel et al., 2013). Two notable exceptions to this trend were found by the authors. In one case, it concerns a study comparing three scales across 26 populations (Hibbing et al., 2019). This study, however, only compares populations in the western hemisphere, and all questions concerned political content, which may limit the generalizability of the study. The second case concerns a meta-analysis of 174 articles to find correlations between ERS and other factors, such as race and intelligence (Batchelor & Miao, 2016). Several correlates of ERS are established, but no information on the general ERS strength or prevalence is provided. While these studies thus provide valuable additions to the ERS literature, it is difficult to gain a general understanding of the prevalence and strength of ERS from current studies. The first aim of the current paper is to address this issue by gathering a wide variety of scales measuring different constructs administered to different populations at different timepoints to gain a general estimate of the prevalence of ERS across time, scales, and populations. Throughout the study, we will utilize data from the Programme for International Student Assessment (PISA) to reach this aim. Naturally, this will limit the generalizability of the current findings somewhat, since questions are administered to ∼15-year-old students, and the context is limited to a low-stakes school environment. Nevertheless, the large selection of timepoints, countries, and scales available makes this data a valuable resource to gain initial insight into the prevalence and strength of ERS, with avenues for future research left open to assess the generalizability of findings beyond the PISA population and scales.

In addition to estimating the prevalence of ERS, we wished to obtain an estimate of the general strength of ERS. When formally operationalizing the strength of ERS, a natural choice is to utilize an IRT model where ERS is modeled in addition to the latent trait. The strength estimate (e.g., the ERS loading when the variance of the ERS dimension is constrained to 1, and the ratio of the substantive loading to the ERS loading) for a given ERS model may inform practice by helping researchers pick realistic conditions for methodological studies, since guidance on this topic is currently lacking (Schoenmakers et al., 2026). Since a wide plethora of these models have been developed in the literature, we will discuss these below. As a third aim, we were interested in which ERS model would generally be preferred in a variety of empirical settings. We investigated this by fitting several ERS models and comparing their fit to each other.

The rest of this paper proceeds as follows. First, the “IRT Models for ERS” section describes several approaches to modeling ERS and outlines the model families and specific models used in this paper. Second, the “Method” section outlines the data and methods used in this paper and the outcomes of interest. Third, the “Results” section contains the results from the analyses described in the Methods section. Finally, the “Discussion” section lists practical recommendations based on the results and outlines limitations of the current study and avenues for future research.

## IRT Models for ERS

While a wide variety of IRT models for ERS exist, they differ substantially in how ERS is modeled and the assumptions they make about response styles (for an overview of these models and their differences, see, for example, Bolt & Meng, 2025; Henninger & Meiser, 2020). One of the most important differences between the various models is whether they conceptualize ERS as a categorical or continuous trait.

When ERS is viewed as a categorical latent trait, mixture IRT models are often used. Mixture IRT models combine IRT modeling with latent class analysis, creating different latent classes based on their observed responses (Rost, 1991). Item and person parameters are separately estimated within each class. This allows for differences in item parameters across classes, but assumes homogeneity within each class. For instance, a two-class mixture IRT model might distinguish between an ordinary responding class and an extreme responding class (Austin et al., 2006; Böckenholt & Meiser, 2017). Crucially, all participants in the ordinary responding class are assumed not to be affected by ERS, and all participants in the extreme responding class are affected by ERS to the same extent. While these mixture IRT models are applied in practice, a limitation of models with a categorical view of ERS is their assumption of no variation in item parameters within each class. This may be an oversimplification, as individuals for whom ERS is present could plausibly vary in their ERS tendency (Huang, 2016). Due to this limitation, this paper focuses on models that treat ERS as a continuous rather than a categorical construct.

A wide variety of IRT models conceptualize ERS as a continuous latent variable. These include heterogeneous threshold models (Johnson, 2003), unfolding models (Javaras & Ripley, 2007), extensions of the rating scale model (Jin & Wang, 2014), multidimensional nominal response models (MNRMs) (Bolt et al., 2014; Bolt & Johnson, 2009; Falk & Cai, 2016) and item response tree (IRTree) models (Böckenholt, 2012; Böckenholt & Meiser, 2017; De Boeck & Partchev, 2012; Jeon & De Boeck, 2016; Meiser et al., 2019; Thissen-Roe & Thissen, 2013). These models all introduce an additional continuous latent trait for ERS in addition to the substantive trait the scale is designed to measure.

Although all the models described above can account for ERS, many lack implementations in widely used software packages, limiting their accessibility for applied researchers. Furthermore, not all of them support modeling other response styles besides ERS or allow researchers to estimate correlations between ERS and the substantive trait, reducing their flexibility. Two important exceptions are the MNRM and IRTree model families. Both can be implemented using standard software for multidimensional IRT, such as the R package mirt (Chalmers, 2012), and are used in practice (see e.g., Zhang & Wang, 2020). In addition, they can accommodate a variety of response styles and allow for estimation of the correlation between the substantive trait and the response style dimension (Falk & Cai, 2016; Meiser et al., 2019). For these reasons, the current paper will focus on these two model families when modeling ERS.

Previous research addressed the differences between MNRM and IRTree models when modeling ERS specifically (Schoenmakers et al., 2024). Important differences between the conceptualization and practical impact of ERS were outlined between the two models. In addition, the paper exploratively established the ability of several information criteria (AIC, BIC, SABIC, and HQ) to accurately recover the data-generating model between the generalized partial credit model (GPCM; Muraki, 1997), an MNRM model, and an IRTree model. Note that while comparisons using the AIC, BIC, SABIC, and HQ were shown to be possible, the use of the likelihood-ratio test should be avoided since the MNRM and IRTree models are not nested. Since results from this model comparison approach were promising, we will utilize the same methods to compare the fit of the models considered in this paper.

When utilizing information criteria to compare non-nested models such as the IRTree and MNRM, care should be taken to ensure these comparisons are theoretically valid. Since research has primarily focused on the AIC and BIC in this regard, we discuss these specifically here. The ability of the AIC and BIC to select between non-nested models has been established (see e.g., Vrieze, 2012) if the models are fitted to the same data and have the same dependent variables (Burnham & Anderson, 2002). When selecting between the MNRM and IRTree models in this paper, both conditions are met. Both the IRTree and MNRM models used in this paper are directly fit to the original response data and model the same dependent variables (the ordinal responses), with the pseudo-items only being used to obtain the probabilities of the original ordinal response variables. The use of information criteria to select between IRTree and MNRM models as used in this paper is thus theoretically appropriate and practically supported through a simulation study.

One important note when comparing the fit of models is that specific formulations of MNRM and IRTree models do not necessarily result in an equal number of item parameters. While comparing the fit of non-nested models with a differing number of parameters is possible using the information criteria outlined above, several factors complicate this comparison in the present study. First, comparing models of differing complexity makes it difficult to conclude whether a more complex model fits the data better due to a superior approach to the modeling of ERS, or merely due to the flexibility provided by the extra parameters. In other words, it is possible that the extra flexibility in the model provided by the extra parameter(s) absorbs some kind of model misfit not directly related to ERS. While the model would generally fit the data better in this instance, we should hardly conclude it is a better model for ERS. Second, there is no clear consensus among all four information criteria on how models should be penalized for including more model parameters. As the size of the penalty used will affect which model is preferred, and which penalty should be used is not clear, this introduces a confound when comparing the models.

To alleviate the impact of these potential issues as much as possible, we conducted the comparison of models in two ways. First, we conducted an unconditional comparison of models, where all models are compared directly using the four information criteria outlined above, regardless of the number of item parameters of each model. Second, we conducted the comparison by creating tiers of different ERS models with an equal number of item parameters (in addition to a non-ERS model) and only comparing ERS models that have an equal number of total item parameters to each other. When conducting the analysis in tiers, the models were split into tiers ranging from 1 to 3, with models with fewer total item parameters being placed in lower tiers.

### Tier 1

#### MNRM

As a first model of the MNRM family, we utilize the MNRM adaptation developed by Falk and Cai (2016). In this model, ERS can be modeled using a prespecified scoring matrix reflecting the loading of the response style(s) on categories. The model equation for the MNRM is presented in Equation 1:

(1) $$P\left(Y_{i}=k|\theta \right)=\frac{\exp({\left[a_{i}⊙s_{k}\right]}^{T}\theta +c_{\mathrm{ik}})}{\Sigma_{j=1}^{K}\exp({\left[a_{i}⊙s_{j}\right]}^{T}\theta +c_{\mathrm{ij}})},$$

where $P\left(Y_{i}=k|\theta \right)$ denotes the probability of answering in a certain category k for an item i given the participants ability θ , $a_{i}$ is a vector of item slope parameters, ⊙denotes Schur/Hadamard multiplication, $s_{k}$ is a vector of scoring matrix s , and c denotes a category intercept, with the first intercept fixed to zero. Note that throughout the paper, we will limit ourselves to four-category data. To model ERS for a four-category item, s can be defined as in Equation 2:

(2) $$\left[\begin{array}{c} 0\;1\;2\;3\\ 1\;0\;0\;1 \end{array}\right].$$

The first row of this scoring matrix relates to the substantive dimension, with the second row relating to the ERS dimension. Note that if the ERS dimension is removed from the model, the MNRM simplifies to a generalized partial credit model (GPCM; Muraki, 1997). In total, this adaptation of the MNRM uses 5 item parameters per item: a slope for the substantive trait, a slope for the ERS trait, and 3 item intercepts.

#### IRTree

**Figure 1.**
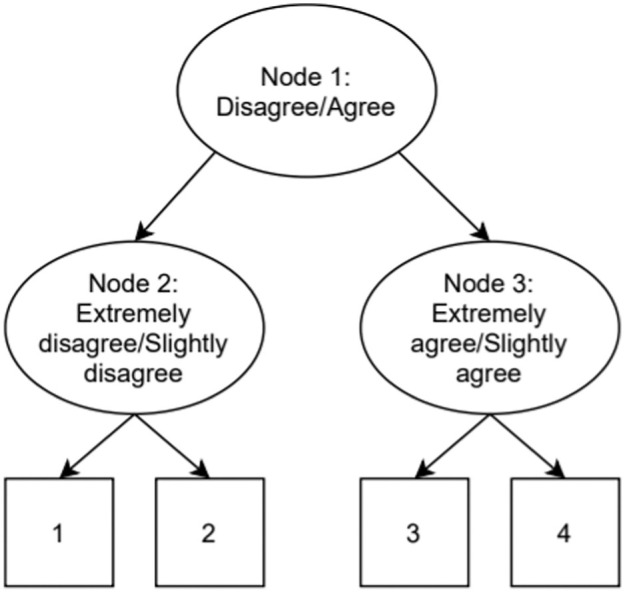
Example of an IRTree Decision Process for a 4-Category Item.

As a first model from the IRTree family, we utilized a multidimensional node IRTree model (Meiser et al., 2019; Schoenmakers et al., 2024; Thissen-Roe & Thissen, 2013). In the model used here, a four-category item is split into three nodes/pseudo-items as depicted in Figure 1.

A general equation for an IRTree node with both a substantive loading and an ERS loading was provided by Schoenmakers et al. (2024). This equation is presented in Equation 3:

(3) $$P(Y_{\mathrm{im}}=1|\theta )=\frac{\exp(\sum_{v=1}^{2}\alpha_{\mathrm{imv}}\theta_{v}+d_{\mathrm{im}})}{1+\exp(\sum_{v=1}^{2}\alpha_{\mathrm{imv}}\theta_{v}+d_{\mathrm{im}})},$$

where $\alpha_{\mathrm{imv}}$ denotes the slope parameter of item *i* in node *m* for dimension *v*, $\theta_{v}$ denotes the *v*^th^ latent trait, with the first dimension being the substantive trait and the second dimension being the ERS trait, and $d_{\mathrm{im}}$ denotes the intercept of item *i* in node *m*. When leaving all parameters unconstrained, 9 item parameters are estimated (3 substantive slopes, 3 ERS slopes, and 3 node intercepts). In Schoenmakers et al. (2024), three constraints were placed on the parameters to identify the model and enable the estimation of a correlation between ERS and the substantive trait. First, the ERS slope in node 1 was constrained to zero, as ERS conceptually should not have an impact on the probability of agreeing with an item (i.e., scoring a 3 or 4). Second, the ERS slope was set to be opposite across node 2 and node 3. Finally, the substantive slope was set to be equal across node 2 and node 3.

Note that if these constraints are used, they result in 9−3=6 estimated parameters per item. To facilitate comparisons with the MNRM, which only has 5 parameters per item, we imposed an additional constraint. The less-constrained IRTree model presented in Schoenmakers et al. (2024) will instead be used in tier 3. In the first tier, we constrained the substantive slope in nodes 2 and 3 to be equal to the substantive slope in node 1, resulting in 5 parameters per item. These parameters are comparable to the MNRM: 1 slope for the substantive dimension, 1 slope for the ERS dimension, and three item intercepts. Throughout the paper, the model with the additional constraint will be referred to as the IRTree $\alpha_{1}$ . In addition to constraining the IRTree model by Schoenmakers et al. (2024), we also wished to increase the flexibility of the MRNM model by Falk and Cai (2016) to match that of the original IRTree model provided by Schoenmakers et al. (2024) while providing IRTree-based models with a matching number of item parameters. The following tiers work toward this goal.

### Tier 2

#### MNRM

To somewhat increase the flexibility of the MNRM in modeling the substantive dimension, an adjustment to the model presented before is proposed. In the first MNRM model, the first row of the scoring matrix s was fixed to [0,1,2,3,4] . We now introduce an additional parameter b , such that the first row of the scoring matrix is [0,b,(3−b),4] . The new b parameter is meant to provide more flexibility for the MNRM on the substantive trait side. This brings this model closer to the IRTree formulation by Schoenmakers et al. (2024), which provides the IRTree with an additional substantive slope set to be equal across nodes 2 and 3. While we freely estimated the b parameter in our study, we advise caution when interpreting models with b values above 2 in practice, since this results in an unordered scoring matrix for the substantive trait. In this tier, we constrain the b parameter to be equal across all items in a scale. We thus obtain 5 parameters that are unique per item, with 1 item parameter being estimated per scale. We refer to this model as the MNRM b .

#### IRTree

To match the added parameter for the MNRM, we now add a proportionality constraint P for the IRTree model. Instead of setting the substantive slopes in nodes 2 and 3 equal to the substantive slope in node 1, the slopes in nodes 2 and 3 are estimated as $\alpha_{i11}*P$ . To mirror the MNRM b , the P parameter is set to be equal across all items in a scale, again resulting in 5 parameters which are unique per item, with 1 parameter estimated per scale. We refer to this model as the IRTree P .

### Tier 3

In the final tier of model comparisons, the flexibility of the MNRM b was increased by estimating the b for each item individually, rather than estimating a single b for the entire scale. This model is referred to as the MNRM $b_{i}$ . After estimating the b parameter to be item-specific, 6 item parameters are estimated, matching the IRTree model as presented by Schoenmakers et al. (2024). We thus use this formulation of the MNRM model as the MNRM model for this tier, and the IRTree model formulated by Schoenmakers et al. (2024) as the IRTree comparison model in tier 3.

## Method

All data and code used for the analyses can be found via https://osf.io/djp25. To estimate the prevalence and strength of ERS over different timepoints, scales, and populations, a large amount of data was needed. The Programme for International Student Assessment (PISA) provides publicly available cross-sectional data gathered worldwide from approximately 15-year-old student populations. The PISA data were gathered from 2000 to 2022 in 3-year intervals (except the 2021 timepoint, which was gathered in 2022 instead), and each year contains a variety of scales (Adams & Wu, 2003; OECD, 2005, 2009, 2010, 2012, 2014, 2017, 2021, 2024). Typically, the questionnaire is split into a cognitive part measuring math, reading and science skills, and a large non-cognitive part measuring various demographic variables and general attitudes. Since we are interested in response styles, we focus on the non-cognitive part of the questionnaire administered to students here.

Several exclusion criteria were applied before any data was analyzed. First, we were specifically interested in 4-category scales. In addition, the scales had to be Likert-type scales clearly measuring a latent construct (i.e., the items capturing the extent to which a participant agreed with a statement or felt a certain way). Forty-seven of these scales were identified in the PISA datasets, with scales, for example, measuring math anxiety or reading enjoyment. Example items from the math anxiety scales include “I often worry that it will be difficult for me in Mathematics classes”, and “I get very tense when I have to do Mathematics homework”, scored from 1 (“strongly agree”) to 4 (“strongly disagree”). All 47 scales identified were answered by 40 to 80 populations (depending on the year of administration), resulting in 2,960 scale-population pairs, which were considered. A single scale administered to a single country in a single year will be referred to as a dataset throughout the paper.

Several further exclusion criteria based on preliminary analysis were used. First, we wished to obtain reasonable power to detect ERS if it was present. To this end, we only retained datasets where at least 1,000 participants answered at least one item in the scale (677 exclusions; 643 of these exclusions happened in the 2022 dataset. This is due to the use of a rotated design in that year, where not every scale was administered to every participant). In addition, we removed datasets where all participants agreed with more than 90% or less than 10% of items (128 exclusions), since these datasets will provide relatively little information on the true standing of participants on the latent trait and may lead to convergence issues or high standard errors for the estimated parameters. Finally, we conducted a rudimentary check for unidimensionality. While technical manuals for all datasets were consulted to ensure datasets were at least intended to be unidimensional, unintended multidimensionality may occur in practice. This multidimensionality could then affect the study outcomes. Note that the topic of assessing strict or approximate unidimensionality in datasets is not straightforward, and many methods have been proposed (Slocum-Gori & Zumbo, 2011; Ziegler & Hagemann, 2015). In our paper, we resolved to use a relatively simple check for approximate unidimensionality. We calculated the eigenvalues of the polychoric correlation matrix between the items for each dataset. If the first eigenvalue was not at least 4 times greater than the second eigenvalue (see, for example, Humphris et al., 2018 for an application of this rule), the scale was discarded (698 exclusions). Note that while we cannot conclude with full confidence that all remaining datasets are approximately unidimensional, it is likely that many strongly multidimensional datasets have been removed. Applying the exclusion criteria mentioned above left 38 out of 47 scales, with 1,457 out of 2,960 original datasets retained for further analysis. Supplementary material A lists a more complete overview of the datasets, their topics, which datasets were eliminated, and how many populations remained for each scale.

To address the first aim of the study (general prevalence of ERS), a comparison was made between the ERS models and a non-ERS model. As a non-ERS model, we chose to utilize the GPCM. The GPCM was chosen as a non-ERS model in this study for several reasons. First, the original PISA data were analyzed using the partial credit model from 2000 to 2012, while later years utilized a GPCM. While not conclusive evidence, it is somewhat likely that questionnaires designed to be analyzed using a (G)PCM would fit a (G)PCM relatively well compared to other non-ERS models. Second, the GPCM has a clear relationship to the MNRM model used in this study, since removal of the ERS dimension in the MNRM results in a GPCM. Finally, the GPCM is a commonly used IRT model that practitioners are likely to be familiar with.

The IRTree family of models, the MNRM family of models and the GPCM model were fit to all 1457 datasets using the mirt R package (Chalmers, 2012) with marginal maximum likelihood estimation. Standard IRT identification constraints were used, such that the mean of every latent variable was fixed to zero and the variance of every latent variable was fixed to 1. For each dataset, correlations between the substantive trait and the ERS trait were freely estimated. For every model, the AIC, BIC, SABIC and HQ were calculated; four information criteria were calculated by default in mirt. Recall that previous research has established the ability of these specific information criteria to recover the data-generating model between a GPCM, MNRM, and IRTree model under simulation conditions (Schoenmakers et al., 2024).

### Outcomes

To answer the first research question regarding the prevalence of ERS, the fit of the ERS models and the GPCM were compared once in every tier (with every tier containing the GPCM and two ERS models) and again in a non-tiered comparison using the information criteria. The model preferred by most information criteria was chosen as the preferred model, with any ties between models broken randomly. If any of the ERS models were preferred over the GPCM, this was taken to indicate the presence of ERS in the data.

To answer the second research question, the item parameters of all estimated models were saved. Note that while all parameters were saved, it is not straightforward which of these parameters would be of interest to a researcher. On the one hand, they may be interested in the model parameters regardless of which model was preferred by the information criteria, since this will give the best indication of what model parameters researchers are likely to find when they apply a certain model to empirical data. On the other hand, they may be interested in the model parameters only in cases where that model is actually preferred by the information criteria, since this will give the best indication of what the model parameters are likely to be in cases where the model fits the data best. In addition, models that were not preferred by the information criteria may suffer from some kind of model misfit, which could lead to their parameters being biased.

To avoid the potential bias resulting from displaying model parameters that were not preferred by the information criteria, we chose not to display these in the main paper. An exception to this rule is more complex versions of simpler models, since these complex models simplify to the simpler model if a freely estimated parameter is instead constrained to a certain value. These complex models are thus not only not affected by misfit when a simpler model is preferred, but the exclusion of cases where a simpler model is preferred would result in certain parameter values (i.e., parameter values which are close to the value the parameter is constrained to for the simpler model) not being shown. Not showing these parameters for the more complex model may then mislead the reader into believing these values do not occur. We thus display the subset of model parameters where a model or a simpler version of that model is unconditionally preferred by the information criteria.

Since we only display a subset of all model parameters, it is possible that selection effects (the parameters in the subset of selected model parameters differ substantially from the model parameters if no selection is made) occur. Since these selection effects may be of interest to researchers who believe the parameters of models not preferred by the information criteria still contain valuable information, we display the parameters for all models regardless of preference in supplementary material B. In this supplementary material, we also contrast these findings with the findings presented in the main paper. In addition, supplementary material C contains an explicit comparison of how model parameters of the most complex MNRM and IRTree models differ depending on whether an IRTree or MNRM family of model was preferred, which grants further insight into possible selection effects and may again be of interest to the aforementioned researchers.

While several model parameters are presented, the ERS slope and the ratio of the ERS slope to the substantive slope are of primary interest when considering the strength of ERS. In addition to the item parameters themselves, the correlations between various item parameters and the correlation between ERS and the substantive trait may also be of interest and are thus presented. Note that when presenting the parameters, we chose to present the natural logarithm of the slopes rather than the slopes themselves. Since Pearson’s r would be affected by this transformation (i.e., the correlation between the natural logarithm of the slopes is not the same as the correlation between the untransformed slopes), we instead opted to utilize Kendall’s τ when calculating the correlation between various model parameters. Similar to Pearson’s r , values of one indicate a strong positive association between the rank of variables (as one variable increases in rank, the other variable also increases in rank), values of minus one indicate a strong negative association between the rank of variables, and values close to zero indicate the ranks of variables are not related.

To answer the third and final research question, we wished to gain an overview of which model fits the data most often. This was achieved by comparing the AIC, BIC, SABIC and HQ for all ERS models, both within each tier and in a non-tiered fashion. In case of ties, they were again broken randomly.

## Results

First, results from the tiered comparisons are presented in Table 1. In tier 1, the GPCM model was not preferred in any of the 1457 datasets. Concerning the ERS models, the IRTree $\alpha_{1}$ model was preferred over the MNRM in 74.5% of datasets.

**Table 1.** Tiered Model Preference Counts and Percentages.

Tier 1	Model	GPCM	IRTree $\alpha_{1}$	MNRM
	Count	0	1,086	371
	Percentage	.0%	74.5%	25.5%
Tier 2	Model	GPCM	IRTree P	MNRM b
	Count	0	1,184	273
	Percentage	.0%	81.3%	18.7%
Tier 3	Model	GPCM	IRTree	MNRM $b_{i}$
	Count	3	1,180	274
	Percentage	.2%	81.0%	18.8%

In tier 2, the trend of IRTree family models being generally preferred over MNRM models in a large majority of cases continued and even strengthened. Again, the GPCM was never preferred over the ERS models.

In tier 3, IRTree models were again generally preferred over MNRM models. In addition, we observed the first few cases where the GPCM is preferred over the ERS models. This was likely caused by the large gap in complexity between the GPCM and the ERS models in this tier, since the information criteria balance fit and complexity of models. The large difference in complexity (i.e., number of item parameters) between the models may thus cause some cases to swing toward the GPCM in the absence of other, simpler ERS models. In any case, the IRTree models generally fit the data better than the MNRM models when the number of item parameters is equal.

Besides the tiered comparisons, the non-tiered comparison between models may be of interest. This comparison is presented in Table 2. Several trends become visible. First of all, the GPCM was never the preferred model in any of the 1,457 datasets. Second, the IRTree models were once again generally preferred over the MNRM models in most cases (80.5% vs 19.5%). Third, more complex models (i.e., models with more parameters) were generally preferred more often than models with fewer parameters. Even so, the least preferred IRTree model (i.e., its simplest version) was still preferred more often than the most preferred MNRM model (i.e., the MNRM $b_{i}$ ). The general preference for the IRTree over the MNRM models can thus not be dismissed as merely being related to the complexity of the models.

**Table 2.** Non-Tiered Model Preference Counts and Percentages.

Model	GPCM	IRTree $\alpha_{1}$	IRTree P	IRTree	MNRM	MNRM b	MNRM $b_{i}$
Count	0	184	267	723	63	66	154
Percentage	.0%	12.6%	18.3%	49.6%	4.3%	4.5%	10.6%

### Parameter Estimates

To gain more information about realistic conditions and quantify the strength of ERS, we present the item parameter estimates and the estimated correlations between ERS and the substantive trait. Recall that the parameter estimates shown here were based on cases where the models were preferred over all other models or the models where a simpler case of a more complex model was preferred in the non-tiered comparison, since the parameters are otherwise likely to be inaccurate due to model misfit. Parameters across different models are thus not based on the same number of datasets. We do not present any figures for the GPCM, since it was never preferred in any non-tiered comparison.

**Figure 2.**
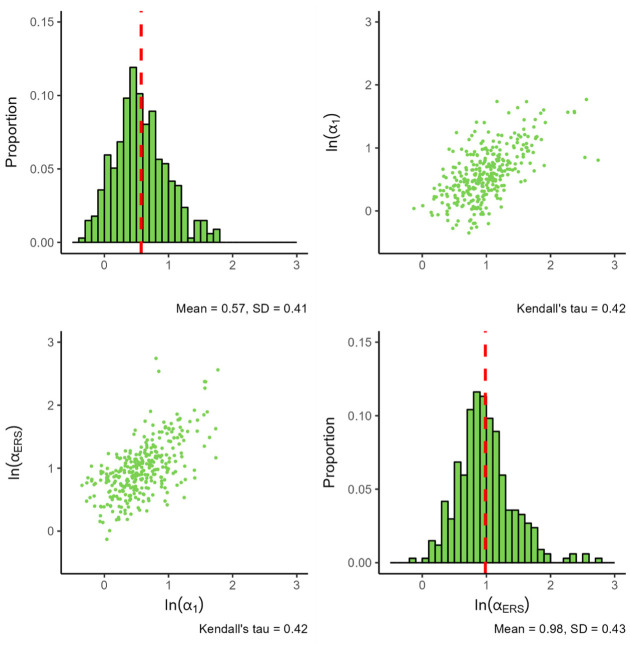
Parameters for the MNRM. *Note. $\ln(\alpha_{1})$* denotes the natural logarithm of the substantive trait loading, and $\ln(\alpha_{\mathrm{ERS}})$ denotes the natural logarithm of the ERS loading. The red dashed line indicates the mean value of each parameter. All plots are based on 316 item parameters from 63 datasets, with each histogram bin being set to a width of 0.1.

Figure 2 presents the results for the MNRM. $\ln(\alpha_{1})$ appears somewhat normally distributed. Interestingly, the mean log of the substantive slope appears far lower than the mean log of the ERS slope. Note that this does not necessarily imply ERS has more impact on the response process than the substantive trait, since the two traits use different scoring matrices in the MNRM.

The standard deviation of both slopes appears similar, although the ERS slope tends to display more extreme values. Nevertheless, the observed mean difference seems better explained by the peak of the ERS slope distribution being at a higher value than the peak of the substantive slope distribution. When examining the association between the substantive and ERS slopes, we see a somewhat linear positive dependence between the two slopes.

**Figure 3.**
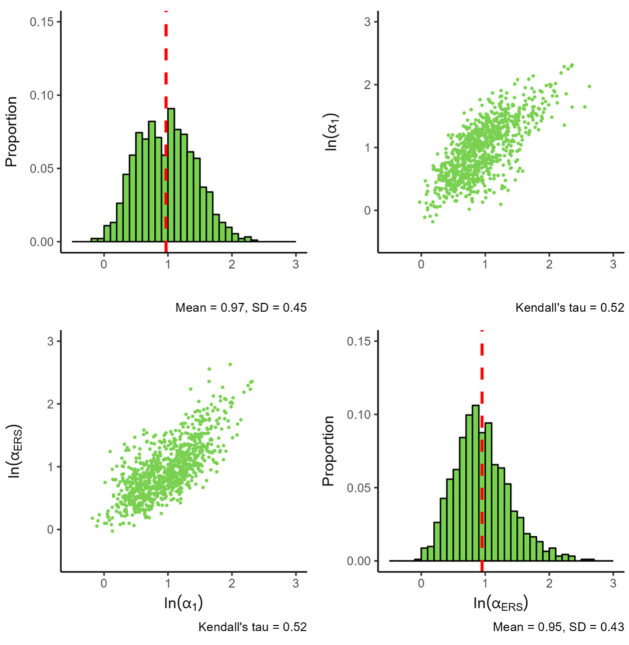
Distribution of Estimated Parameters for the IRTree $\alpha_{1}$ . *Note. $\ln(\alpha_{1})$* denotes the natural logarithm of the substantive trait loading, and $\ln(\alpha_{\mathrm{ERS}})$ denotes the natural logarithm of the ERS loading. The red dashed line indicates the mean value of each parameter. All plots are based on 914 item parameters from 184 datasets, with each histogram bin being set to a width of 0.1.

Figure 3 presents the parameters of the IRTree $\alpha_{1}$ . In the figure, we see that the IRTree $\alpha_{1}$ seems to result in substantive and ERS slopes that are about equal in magnitude, unlike the MNRM. While this is an interesting finding, note that both the magnitude of the slopes and the ratio of the slopes between the IRTree and MNRM models are not directly comparable, since the MNRM directly models a polytomous item while the IRTree instead splits the response process up into binary decision nodes.

The standard deviation of both parameters appears comparable, although the distribution of the ERS log slopes appears a bit more peaked and skewed. Notably, the dependence between the slopes found earlier in the MNRM model persists, with the seemingly linear association between the log slopes increasing in magnitude (0.52 vs. the earlier 0.42).

**Figure 4.**
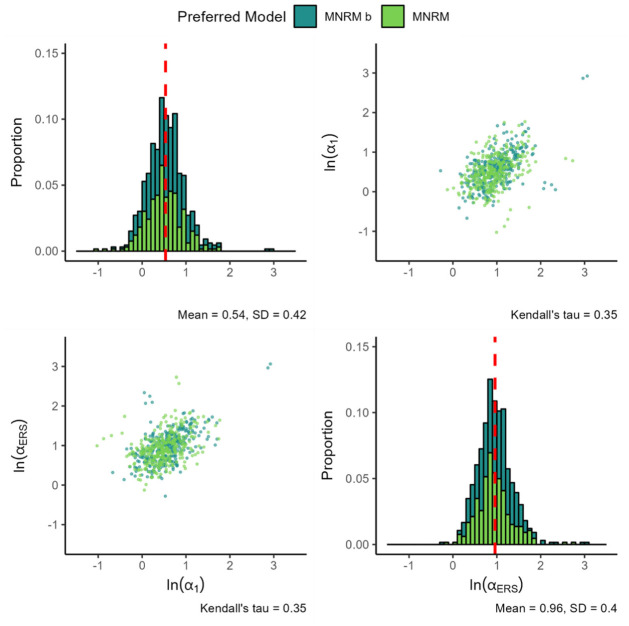
Parameters for the MNRM b . *Note. $\ln(\alpha_{1})$* denotes the natural logarithm of the substantive trait loading, $\ln(\alpha_{\mathrm{ERS}})$ denotes the natural logarithm of the ERS loading, and b denotes the value of the b parameter. The color of the plotted values indicates which model was preferred by the information criteria. The red dashed line indicates the mean value of each parameter. All plots are based on 662 item parameters from 125 datasets, with each histogram bin being set to a width of 0.1, with histogram bars stacked on top of each other.

Figure 4 presents the findings for the substantive and ERS slope of the MNRM b . Note that in this figure, both cases where the regular MNRM was preferred and cases where the MNRM b was preferred, and are included, since the MNRM b simplifies to the MNRM if b is one. In Figure 4, we see that the substantive trait and ERS loadings appear similar to the earlier MNRM model. Notably, the substantive slope remains far lower than the ERS slope. The substantive and ERS slopes remain positively associated, although the magnitude of this association decreases somewhat compared to the earlier models. The magnitude of the association and the values of the log slopes do not seem to overly depend on whether the MNRM or MNRM b was preferred in a given dataset.

**Figure 5.**
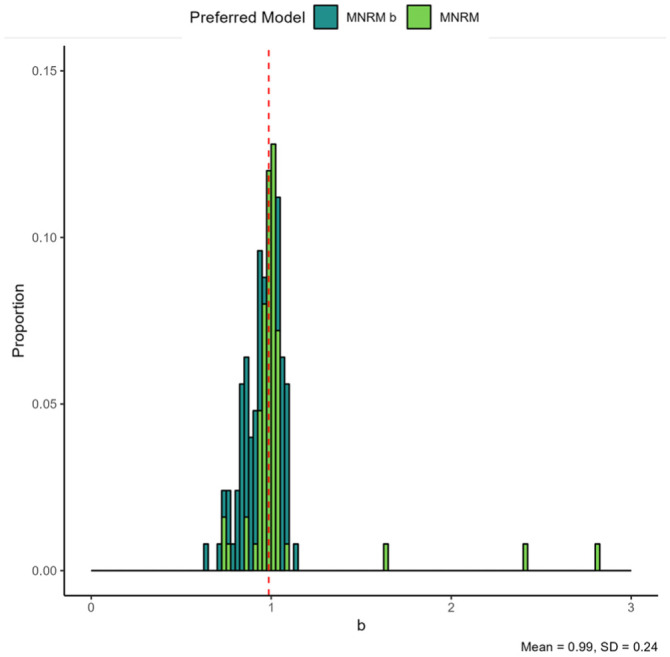
b Parameter for the MNRM b . *Note.* The color of the plotted values indicates which model was preferred by the information criteria. The red dashed line indicates the mean. The plot contains 125 parameters from 125 datasets, with each histogram bin being set to a width of 0.025, and histogram bars stacked on top of each other.

Results for the scale-specific b parameters are presented in Figure 5. In datasets where the MNRM is the preferred model, the b parameter is frequently close to 1. This is to be expected given the nested nature of the models. Some notable outliers were relatively extreme b values occur despite a preference for the MNRM to occur. Further inspection of these cases revealed a strong asymmetry in responses, with lower categories (1 and 2) being endorsed about 7 times less often than higher categories (3 and 4). This may explain this phenomenon. Notably, values of b below 1 seems to occur more than values of b above one in cases where the MNRM b was preferred.

**Figure 6.**
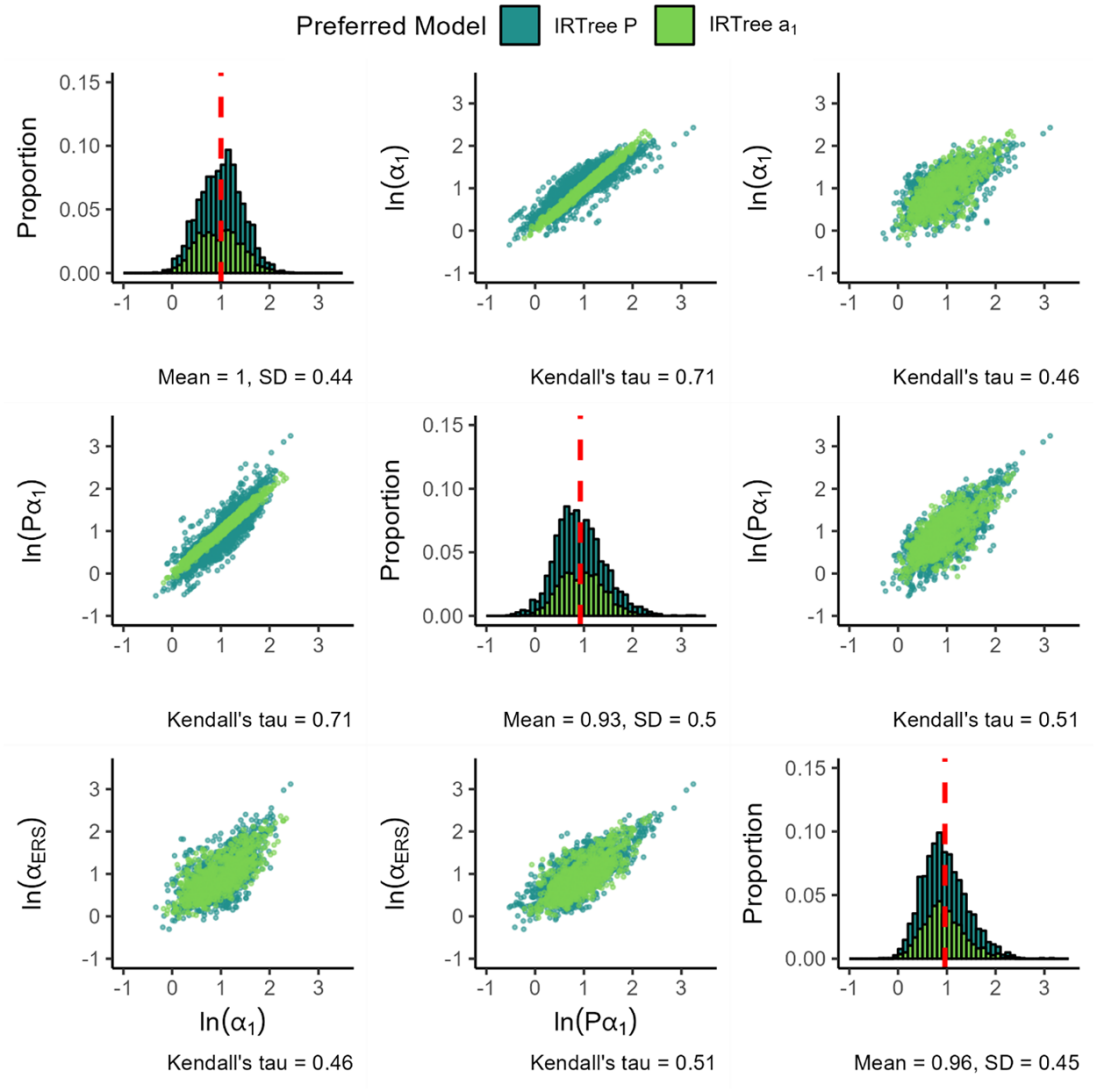
Item Parameters for the IRTree P . *Note. $\ln(\alpha_{1})$* denotes the natural logarithm of the substantive trait loading, $\ln(P\alpha_{1})$ denotes the natural logarithm of the substantive slope in nodes 2 and 3 (calculated as $P*\alpha_{1}$ ), $\ln(\alpha_{\mathrm{ERS}})$ denotes the natural logarithm of the ERS loading, and P denotes the value of the P parameter. The color of the plotted values indicates which model was preferred by the information criteria. The red dashed line indicates the mean value of each parameter. Plots are based on 2219 item parameters from 450 datasets, with each histogram bin being set to a width of 0.1 and histogram bars stacked on top of each other.

Figure 6 displays the parameters of the IRTree P model. For this model, the spread of the ERS slope seems somewhat higher than the spread of the substantive slope. The substantive and ERS slopes remain positively associated in a linear fashion.

Note that the IRTree P model offers the possibility to compare the strength of the substantive and ERS trait loadings in nodes 2 and 3. To facilitate this comparison, we additionally plot $P*\alpha_{1}$ (equivalent to the substantive trait loading in nodes 2 and 3 in this model) and its correlation with the other loadings. In general, the substantive trait loading in nodes 2 and 3 is somewhat lower than the loading in node 1, while being very slightly lower than the ERS loading in nodes 2 and 3. The correlation with both the loading in node 1 and the ERS loading in nodes 2 and 3 is high. In the scatterplots of $P\alpha_{1}$ and $\alpha_{1}$ , we see a strong preference for the IRTree $\alpha_{1}$ model over the IRTree P model along the identity line. This is to be expected, given that the IRTree P model simplifies to the IRTree $\alpha_{1}$ model in cases where P=1 , and if P=1 , $P\alpha_{1}=\alpha_{1}$ .

**Figure 7.**
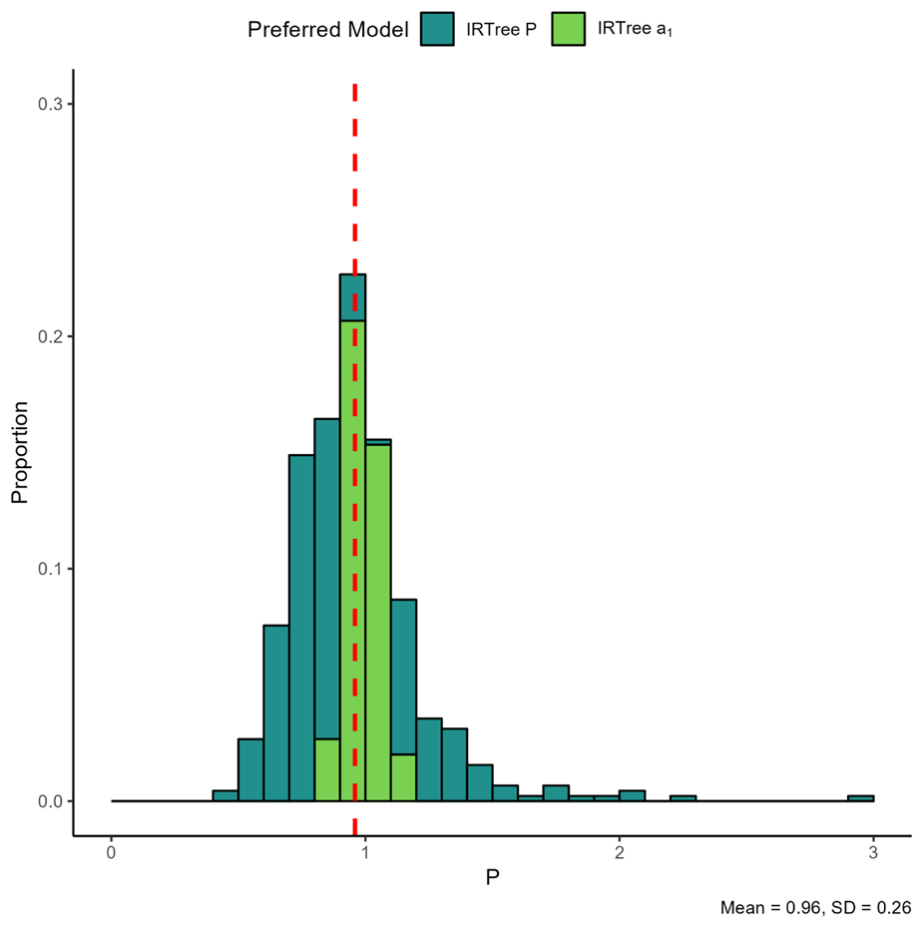
Item Parameters for the IRTree P . *Note.* The color of the plotted values indicates which model was preferred by the information criteria. The red dashed line indicates the mean value of the parameter. The plot is based on 450 scale parameters from 450 datasets, with each histogram bin being set to a width of 0.1 and histogram bars stacked on top of each other.

Figure 7 displays the P parameter for the IRTree P . Again, note that in cases where the IRTree $\alpha_{1}$ was preferred, the P value is always very close to one. When the IRTree P is the preferred model, the P value tends to be between .5 and 1.5, with some outliers on the positive end indicating cases where the substantive trait loading in nodes 2 and 3 was far higher than that in node 1. Notably, the P parameter seems somewhat more “well-behaved” than the MNRM b ’s b parameter in the sense that the IRTree $\alpha_{1}$ is only ever preferred when the P parameter is close to 1, unlike the earlier plots of the b parameter where the MNRM was still preferred over the MNRM b in some cases where the b parameter was not close to 1.

**Figure 8.**
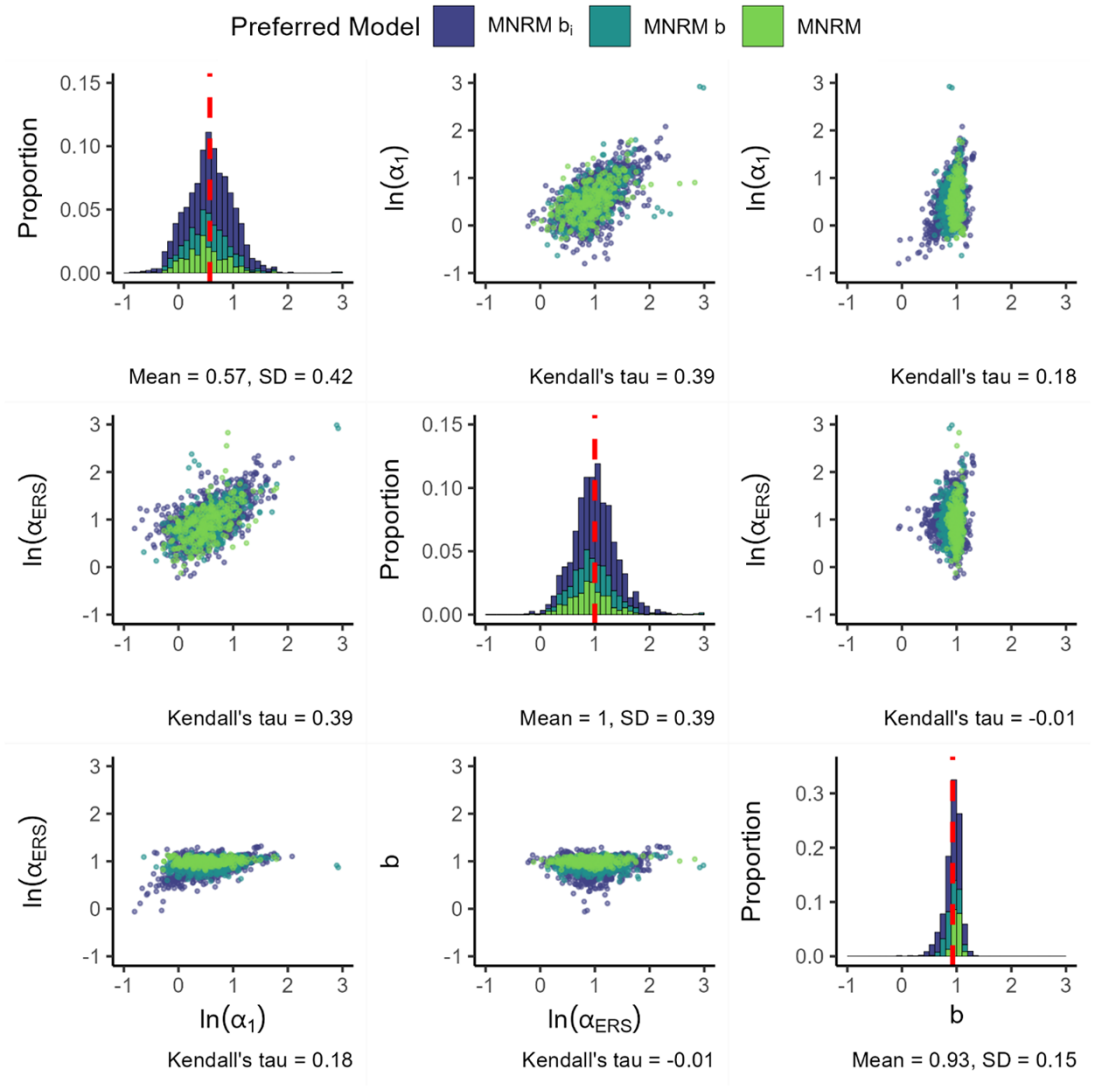
Log Loadings for the MNRM $b_{i}$ . *Note. $\ln(\alpha_{1})$* denotes the natural logarithm of the substantive trait loading, $\ln(\alpha_{\mathrm{ERS}})$ denotes the natural logarithm of the ERS loading, and $b_{i}$ denotes the value of the $b_{i}$ parameter. The color of the plotted values indicates which model was preferred by the information criteria. The red dashed line indicates the mean value of each parameter. Plots are based on 1486 item parameters from 266 datasets, with each histogram bin being set to a width of 0.1 and histogram bars stacked on top of each other.

For the final MNRM-based model, Figure 8 presents the parameters for the MNRM $b_{i}$ . In general, the substantive and ERS slopes are similar to previous MNRM models. When considering the $b_{i}$ parameter, we see a large peak around 1. This indicates most individual items show a decent fit to the regular MNRM (where the b parameter is one), even though the MNRM $b_{i}$ is preferred at the scale level (likely due to items in a scale not sharing the same $b_{i}$ parameter). The log of the substantive and ERS slopes is again positively associated. Note that while the substantive slope and the $b_{i}$ parameters are also somewhat associated; there is virtually no association between the log ERS slope and the $b_{i}$ parameter.

**Figure 9.**
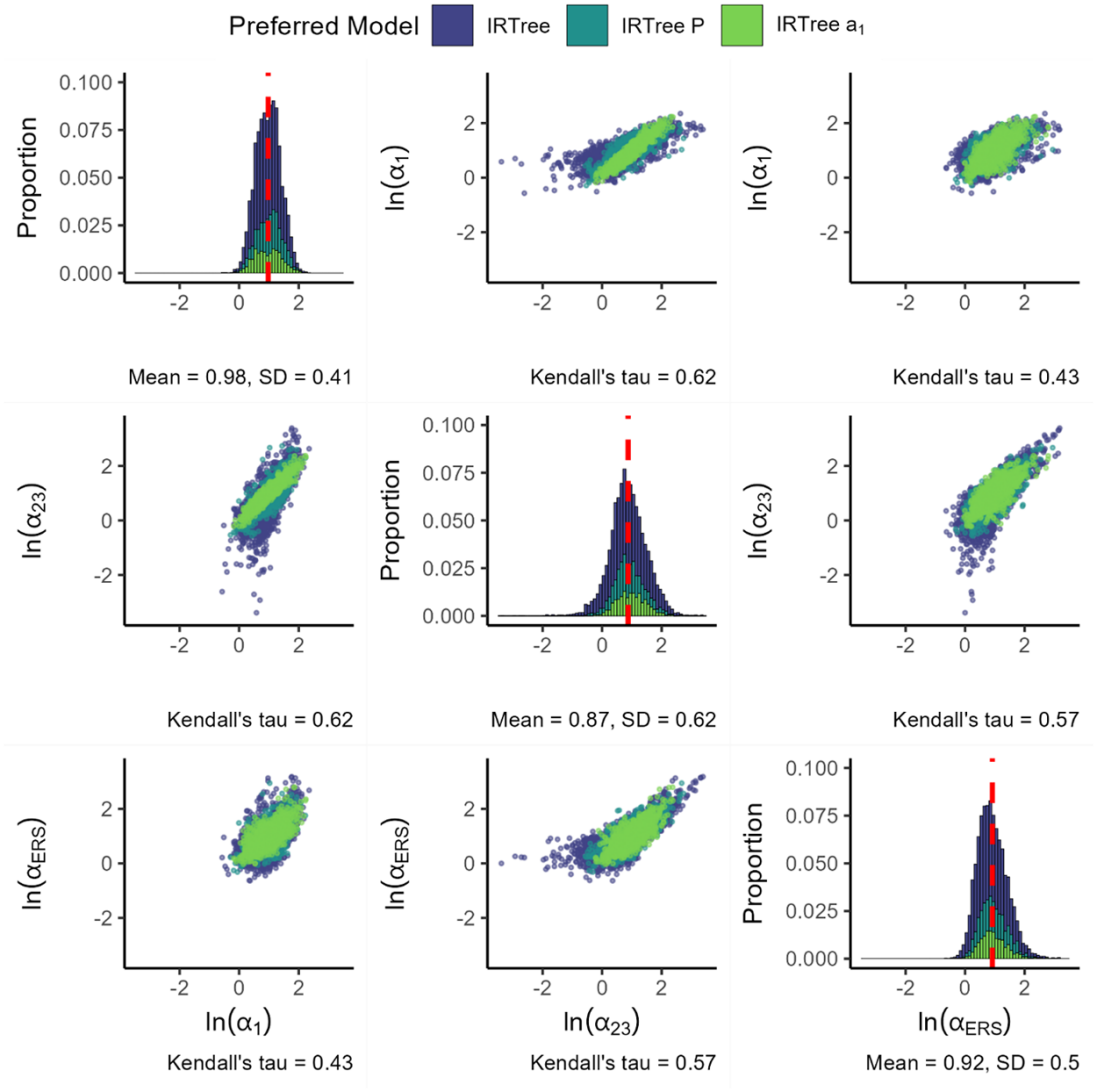
Parameters for the IRTree. *Note. $\ln(\alpha_{1})$* denotes the natural logarithm of the substantive trait loading, $\ln(\alpha_{2/3})$ denotes the natural logarithm of the substantive slope in nodes 2 and 3 and $\ln(\alpha_{\mathrm{ERS}})$ denotes the natural logarithm of the ERS loading. The color of the plotted values indicates which model was preferred by the information criteria. The red dashed line indicates the mean value of each parameter. All plots are based on 6,234 item parameters from 1,165 datasets, with each histogram bin being set to a width of 0.1.

Figure 9 presents the loadings for the IRTree as the final model in the IRTree family. Findings for the substantive and ERS slopes are largely identical to the previous IRTree models. When freely estimating the slope in nodes 2 and 3, we find that it is generally lower than the substantive slope in node 1. In addition, the slope in nodes 2 and 3 appears quite a bit more variable than the other two slopes, both when considering the standard deviation and the range of values that appear in practice. The magnitude of the ERS slope is somewhat higher than that of the node 2 and 3 substantive slope, but lower than that of the node 1 substantive slope.

When considering the associations between the variables, we observe relatively strong positive associations between the three item slopes. The strongest association occurs between the substantive slope in node 1 and the substantive slope in node 2 and 3, followed by the association between the substantive slope in node 2 and 3 and the ERS slope and finally the association between the substantive slope in node 1 and the ERS slope. Note that in cases where the node 2 and 3 substantive slopes are close to the node 1 substantive slope, the IRTree $\alpha_{1}$ is usually preferred.

**Figure 10.**
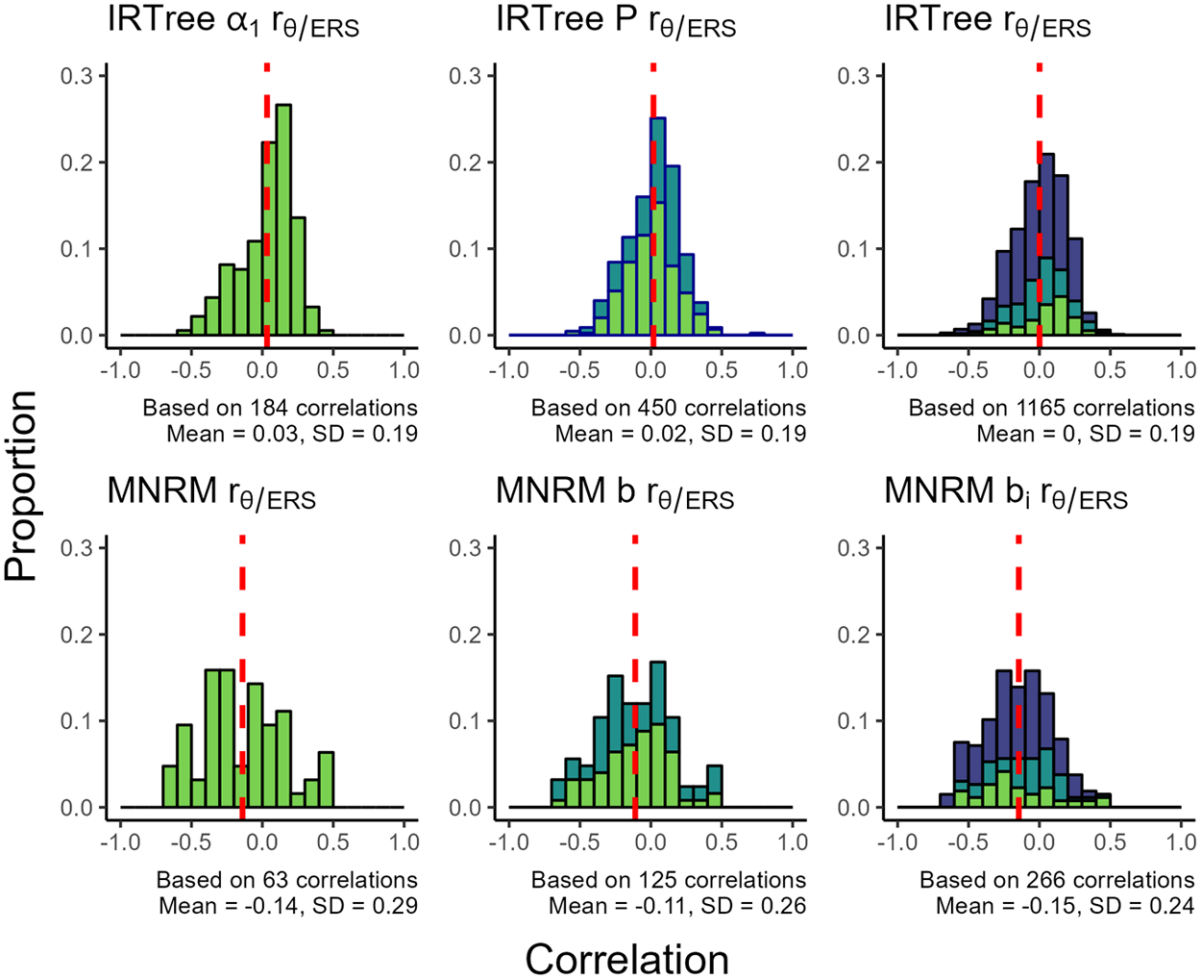
Proportion of Correlations Between ERS and the Substantive Trait With a Certain Value for the Various Models. *Note. $r_{\theta /\mathrm{ERS}}$* denotes the correlation between the substantive trait and ERS. The color of the plotted values indicates which tier of models was preferred by the information criteria, with light green indicating a preference for tier 1, teal indicating a preference for tier 2, and dark blue indicating a preference for tier 3. The red dashed line indicates the mean value of each parameter, with each histogram bin being set to a width of 0.1 and histogram bars being stacked on top of each other.

Finally, Figure 10 presents the correlation between ERS and the substantive trait under the various models. In the figure, we see that the IRTree and MNRM-based models tend to result in somewhat different correlations. Under the IRTree models, the average correlation between the substantive trait and ERS is near (or at) zero. Under the MNRM model, the average correlation is negative. Results from supplementary materials B and C indicate this is likely due to a selection effect, where the MNRM models are more frequently preferred in datasets where the correlation is negative. The “correct” set of correlations to generate from for the MNRM then depends on which perspective the researcher holds (should we generate data from a population of datasets where the MNRM is the preferred model, or should we generate data from an unconditional population of datasets?).

When considering the distribution of correlations, the IRTree models tend to produce somewhat of a bell-shaped distribution. The distribution of correlations under the MNRM models tends to be less peaked than the IRTree models, especially for the simpler MNRM models, which were preferred less often. This is reflected in the larger SD for the estimated correlations under the MNRM models compared to the IRTree models.

## Discussion

The present study set out to address three gaps in the literature. First, we wished to obtain an estimate of the prevalence of ERS. Second, we aimed to obtain an estimate of the strength of this ERS when present to aid future methodological studies in creating realistic conditions. Finally, we wished to compare the fit of various ERS models to assess which ERS model is generally preferred in empirical settings. The research questions will be discussed in this order.

In our study, ERS models were preferred over the non-ERS model in each and every dataset we examined, signaling an extremely high prevalence of ERS. These findings are rather striking given the large number of scales examined across a wide variety of populations and timepoints. Note that while the prevalence estimate seems high, it is in line with earlier research using posterior predictive checks to assess the presence of ERS in a subset of PISA data (Schoenmakers et al., 2025) and research utilizing PCM mixture models identifying preferences for a two-class solution related to ERS in many facets of the NEO-PI-R (Wetzel, Böhnke, et al., 2013) and in PISA data from 2006 (Wetzel, Carstensen, et al., 2013). The high prevalence of ERS indicates it could be a widespread source of bias in questionnaires, and measures to correct for its influence should be considered.

When considering the modeling of ERS, researchers may be hesitant out of fear that response style factors could absorb true variance from substantive traits, or that the substantive trait mean could be biased after implementing an ERS dimension. Note that while previous research has shown the potential for unidimensional ERS IRTree models to capture part of the substantive trait variance in conditions where no ERS is present, this effect was not found for multidimensional node IRTree models as used in this paper (Merhof et al., 2024). In addition, other research also finds little to no bias in the substantive trait mean and variance when fitting an MNRM or a multidimensional node IRTree model in conditions where no ERS is present (Schoenmakers et al., 2024). Combined, this research suggests that not fitting an ERS model in cases where ERS is present could be a far more impactful mistake than applying an ERS model in cases where ERS is not present. Researchers would do well to take these findings into account when considering the modeling of ERS.

To examine the strength of ERS, item parameters for the ERS were saved and presented. By presenting the full distribution of parameters, our study established a realistic spread of item parameters for the various models. A first interesting finding here was the discrepancy between the substantive and ERS slope relationship under both models. Under IRTree models, the slope of the ERS dimension was, on average, roughly equal to the slope of the substantive dimension. Under MNRM models, the substantive slope was, on average, far lower than the ERS slope. While this finding may have been partially caused by a selection effect for the MNRM, it persisted even when we did not select for cases where the MNRM was preferred. Future methodological studies would do well to take this discrepancy into account.

A second interesting finding was the correlation between ERS and the substantive trait being negative on average for the MNRM models, while the same correlation was on average around zero for the IRTree models. Further analyses detailed in supplementary material C showed that this effect is likely caused by the information criteria in this study preferring the MNRM family of models in cases where the correlation between the substantive trait and ERS was negative, rather than the MNRM resulting in negative correlations regardless of which model was preferred. Which set of correlations a researcher should generate from will depend on the population of interest: datasets where the MNRM fits best (which are reported in the main paper), or the unconditional set of datasets (which can be found in supplementary material B). The correlation between the response style trait and the substantive trait may be of special interest since earlier research (Plieninger, 2017) found that a non-zero correlation increases the bias resulting from ignoring a response style.

Finally, substantial positive correlations between the slope(s) for the substantive trait and the slopes for the ERS trait were found across all models. This positive correlation between ERS and substantive trait loadings found in this study should be considered when generating data for future simulations, since the correlation between item slopes may affect the results of simulation studies (Schoenmakers et al., 2026).

As a final goal, we compared the ERS models to each other in terms of fit, both independently of the number of item parameters and in a tiered comparison based on a matching number of item parameters. When comparing the various models in terms of fit, regardless of the number of item parameters, there was a large proportion of cases where the IRTree family of models fit the data better than the MNRM family of models. In general, more complex models were preferred over simpler models, with the IRTree model being most popular. Conclusions in the tiered analysis were similar; the IRTree models were preferred far more often than the MNRM models. While the IRTree models were generally preferred far more often than the MNRM models, we do not advise applied researchers to apply only an IRTree model without considering other options, since the MNRM was still preferred in ∼20% of cases. Instead, a comparison of model fit between various ERS models should be conducted. Choice of a model on theoretical grounds (i.e., conceptualization of ERS or the response process) is another valid approach to choosing an ERS model.

The preference for IRTree models observed in our paper could occur for several reasons. First, it could be that the data-generating model more closely resembles an IRTree model in the majority of datasets examined here. One reason for this could be that the node operationalization of the IRTree allows for a cleaner specification of an extreme response style compared to the divide-by-total nature of the MNRM. The fact that these operationalizations result in qualitatively different conceptualizations of ERS has previously been outlined in the literature (Schoenmakers et al., 2024). The current paper reinforces these findings by showing a large difference between the preference for these models, while simultaneously establishing that the IRTree models appear to be preferred more in practice.

Another possibility is that the extra slope parameter in nodes 2 and 3 allows the IRTree somewhat more flexibility than the MNRM, even after the addition of the b parameter in the MNRM b and MNRM $b_{i}$ . This could explain some of the preference, although it does not explain how the IRTree $\alpha_{1}$ was already preferred over the MNRM in ∼75% of cases. The node operationalization of the IRTree thus seems to be the most likely candidate for explaining the discrepancy between models.

While the present study makes several valuable contributions to the literature, several limitations and avenues for future research remain. First, the current study examined only data provided by PISA, which naturally brings along several limitations from this dataset. For example, the questionnaire is relatively long, which may induce survey fatigue. In addition, it only measures 15-year-old students. While students were measured across populations, scales, and timepoints, it may thus be difficult to generalize the findings of this study beyond the student population. Furthermore, we only considered four-category scales. It is likely that scales with a different number of categories will have different ERS and substantive trait loadings. For the MNRM, adding or subtracting categories will require reformulation of the scoring matrix, which will likely affect loadings. For the IRTree, a differing number of categories will require a revision of the tree diagram, which may result in the addition or removal of nodes (and thus also loadings). For these reasons, it would be difficult to straightforwardly compare estimates obtained from items with a different number of categories for either model. Future research would do well to examine the strength and prevalence of ERS in non-student populations and in items with a different number of categories.

Second, the present study only deals with empirical data. While this is a necessity to answer the research questions, the use of empirical data brings many unknown factors with it that may influence the results of the study. In particular, it may be the case that ERS models are preferred not solely due to the presence of ERS, but rather some other type of multidimensionality or GPCM misfit. While we attempted to at least somewhat account for multidimensionality by excluding strongly multidimensional datasets, there is no guarantee that results were not affected by GPCM misfit. Future research would do well to further extend the research into best approaches to test for (approximate) unidimensionality in an IRT context. In addition, the effect of unmodeled multidimensionality on model comparisons between ERS and non-ERS models could be a topic of further investigation.

Third, the current study creates tiers of models based on the number of item parameters in each model. While the idea of comparing models of equal complexity in this manner is appealing, it must be noted that the number of item parameters may be an imperfect measure of model complexity, since models with the same number of item parameters may still differ in their flexibility to accommodate various data patterns. An alternative criterion that could have been used instead of the number of item parameters is the minimum description length approach (see e.g., Myung et al., 2006), which could be used to assess if the MNRM and IRTree models are equally able to flexibly accommodate various possible datasets.

Fourth, the current research design did not allow for a direct comparison of the loading size of the substantive and ERS traits, due to the different scoring matrices utilized. Future research would do well to explore ways to increase the comparability of item slopes, e.g., through the use of normalized vectors of scoring weights. In addition, future research could explore ways to enhance the comparability of item slopes across the MNRM and IRTree models.

Finally, the current paper limited itself to ERS only and considered only some of the possible ERS models. Future research would do well to investigate the prevalence and strength of other response styles, such as the acquiescent response style. Even when only considering ERS as a response style, other models for ERS than the ones considered in this paper could be compared to each other. Note that if other models are to be compared, we encourage researchers to match the number of item parameters of these models as much as possible to avoid this potential confound.

Overall, the current study reveals an incredibly high prevalence of ERS in data originally thought to be unidimensional. Parameter estimates of various ERS models were gathered for future methodological studies, and the IRTree family of models was established as a generally preferred way of modeling ERS based on model fit. We encourage future research to generalize these findings to other datasets and utilize parameters obtained here for simulation conditions.

## Supplemental Material

sj-docx-1-epm-10.1177_00131644261435119 – Supplemental material for How Extreme Is It Anyways?: An Empirical Investigation Into the Prevalence and Strength of Extreme Response Style Supplemental material, sj-docx-1-epm-10.1177_00131644261435119 for How Extreme Is It Anyways?: An Empirical Investigation Into the Prevalence and Strength of Extreme Response Style by Martijn Schoenmakers, Jesper Tijmstra, Jeroen Kornelis Vermunt and Maria Bolsinova in Educational and Psychological Measurement
