# Supplementary material for: How Extreme Is It Anyways?: An Empirical Investigation Into the Prevalence and Strength of Extreme Response Style
Source: Educ Psychol Meas. 2026 Apr 9:00131644261435119. Online ahead of print. doi: 10.1177/00131644261435119 (PMC13068779; doi:10.1177/00131644261435119)
Supplement: sj-docx-1-epm-10.1177_00131644261435119 – Supplemental material for How Extreme Is It Anyways?: An Empirical Investigation Into the Prevalence and Strength of Extreme Response Style [file sj-docx-1-epm-10.1177_00131644261435119.docx]

**Supplementary Material A**

**Details on the data**

**Table A1**

Scales and scale retention for all data

| Scale Characteristics | | | | | |  | Exclusion criteria | | |
| --- | --- | --- | --- | --- | --- | --- | --- | --- | --- |
| Year | Scale | $N_{items}$ | Topic | $N_{total}$ | $N_{kept}$ |  | $N_{eigen}$ | $N_{agree}$ | $N_{1000}$ |
| **2000** | **ST31** | **8** | **Sense of belonging to school** | **42** | **0** |  | **42** | **0** | **0** |
|  | ST35 | 9 | Engagement in reading | 42 | 27 |  | 15 | 0 | 0 |
| *2003* | *ST27* | *6* | *Sense of belonging to school* | *40* | *5* |  | *15* | *19* | *1* |
|  | ST30 1,3,4,6 | 4 | Mathematics enthusiasm and interest | 40 | 40 |  | 0 | 0 | 0 |
|  | ST30 2,5,7,8 | 4 | Motivation to learn math | 40 | 37 |  | 0 | 3 | 0 |
|  | *ST31* | *8* | *Math self-efficacy* | *40* | *18* |  | *22* | *0* | *0* |
|  | ST32 1,3,5,8,10 | 5 | Anxiety in math | 40 | 30 |  | 10 | 0 | 0 |
|  | ST32 2,4,6,7,9 | 5 | Mathematics self-concept | 40 | 39 |  | 1 | 0 | 0 |
|  | *ST34 1,3,4,10,12* | *5* | *Control strategies math* | *40* | *12* |  | *26* | *2* | *0* |
|  | *ST34 2,5,8,11,14* | *5* | *Collaboration strategies math* | *40* | *11* |  | *29* | *0* | *0* |
|  | **ST34 6,7,9,13** | **4** | **Rehearsal strategies math** | **40** | **0** |  | **40** | **0** | **0** |
|  | ST37 1,3,5,7,10 | 5 | Competitive in math | 40 | 36 |  | 4 | 0 | 0 |
|  | *ST37 2,4,6,8,9* | *5* | *Collaborative in math* | *40* | *11* |  | *29* | *0* | *0* |
| 2006 | ST16 | 5 | Enjoyment of Science | 56 | 55 |  | 1 | 0 | 0 |
|  | *ST18 1,2,4,6,9* | *5* | *General value of science* | *56* | *18* |  | *20* | *18* | *0* |
|  | ST18 3,5,7,8,10 | 5 | Personal value of science | 56 | 29 |  | 26 | 1 | 0 |
|  | *ST26* | *7* | *Responsibility for sustainable development* | *56* | *20* |  | *27* | *8* | *1* |
|  | ST29 | 4 | Future-oriented science motivation | 56 | 56 |  | 0 | 0 | 0 |
|  | ST35 | 5 | Instrumental motivation to learn science | 56 | 54 |  | 0 | 2 | 0 |
|  | ST37 | 6 | Science self-concept | 56 | 56 |  | 0 | 0 | 0 |
| 2009 | ST24 | 11 | Enjoyment of reading | 72 | 48 |  | 24/0/0 | 0 | 0 |
| 2012 | ST29 2,5,7,8 | 4 | Instrumental motivation | 64 | 61 |  | 0 | 3 | 0 |
|  | ST29 1,3,4,6 | 4 | Mathematics interest | 64 | 64 |  | 0 | 0 | 0 |
|  | ST37 | 8 | Math self-efficacy | 64 | 43 |  | 20 | 1 | 0 |
|  | **ST43** | **6** | **Perceived control of Mathematics performance** | **64** | **0** |  | **64** | **0** | **0** |
|  | ST46 | 9 | Math work ethic | 64 | 60 |  | 4 | 0 | 0 |
| 2015 | ST034 | 6 | Sense of belonging to school | 73 | 40 |  | 28 | 3 | 2 |
|  | ST094 | 5 | Enjoyment of science | 73 | 72 |  | 0 | 0 | 1 |
|  | ST113 | 4 | Instrumental motivation | 73 | 70 |  | 0 | 2 | 1 |
|  | ST131 | 6 | Epistemological beliefs | 73 | 54 |  | 13 | 5 | 1 |
| 2018 | *ST160* | *5* | *Enjoyment of reading* | *80* | *17* |  | *60* | *0* | *3* |
|  | ST182 | 4 | Working motive and mastery achievement motive | 80 | 40 |  | 38 | 0 | 2 |
|  | ST188 | 5 | Resilience | 80 | 56 |  | 22 | 2 | 0 |
|  | ST034 | 6 | Sense of belonging to school | 80 | 55 |  | 23 | 1 | 1 |
|  | ST204 | 4 | Attitudes towards equal rights for immigrants | 80 | 55 |  | 2 | 4 | 19 |
|  | ST207 | 5 | Perceptions of bullying | 80 | 66 |  | 0 | 12 | 2 |
| 2022 | **ST034** | **6** | **Sense of belonging** | **80** | **0** |  | **4** | **0** | **76** |
|  | *ST265* | *4* | *Feeling safe* | *80* | *9* |  | *65* | *0* | *6* |
|  | *ST263* | *4* | *Growth mindset* | *80* | *32* |  | *0* | *42* | *6* |
|  | *ST290* | *9* | *Formal and applied mathematics* | *80* | *1* |  | *3* | *0* | *76* |
|  | **ST291** | **10** | **Mathematical reasoning and 21^st^ century mathematics** | **80** | **0** |  | **0** | **0** | **80** |
|  | *ST292* | *6* | *Math anxiety* | *80* | *2* |  | *2* | *0* | *76* |
|  | **ST334** | **10** | **Creative thinking self-efficacy** | **80** | **0** |  | **0** | **0** | **80** |
|  | **ST340** | **10** | **Creativity and openness to intellect** | **80** | **0** |  | **0** | **0** | **80** |
|  | ST341 | 5 | Openness to art and reflection | 80 | 58 |  | 15 | 0 | 7 |
|  | **ST342** | **7** | **Imagination and adventurousness** | **80** | **0** |  | **0** | **0** | **80** |
|  | **ST354** | **10** | **Feelings about learning at home** | **80** | **0** |  | **4** | **0** | **76** |

*Note.* Year denotes the year the scale is from, scale denotes the scale name in the PISA data, items denotes the number of items in the scale, topic lists the topic of the scale according to the PISA technical manual, length denotes the number of populations originally answering the scale, retained denotes the number of populations answering the scale after exclusion criteria have been applied, $N_{eigen}$ denotes how often the first eigenvalue was not at least 4 times greater than second eigenvalue, $N_{agree}$ denotes how often there was less than 10% or more than 90% scale agreement, and $N_{1000}$ indicates how often there were less than 1000 participants with at least a single answer to the scale. Entries in **bold** were fully excluded, while entries in *italics* had at least half of the population-scale combinations excluded.

**Supplementary Material B**

**Plots When All Model Parameters Are Shown**

Since researchers may believe the distribution of the model parameters is of interest regardless of which model was preferred by the information criteria, we display these parameters in this supplementary material. Figure B1 contains the parameters for the MNRM regardless of which model was preferred. We see that the mean of the log substantive trait loading increases compared to the case where we only show model parameters when the MNRM is preferred (0.57 vs 0.71).The mean ERS slope is relatively unaffected (0.98 vs 0.96) compared to the substantive slope. In addition, the relationship between the two slopes remains roughly the same (.42 vs .46 tau values).

**Figure B1**

Parameters for the MNRM regardless of model preference


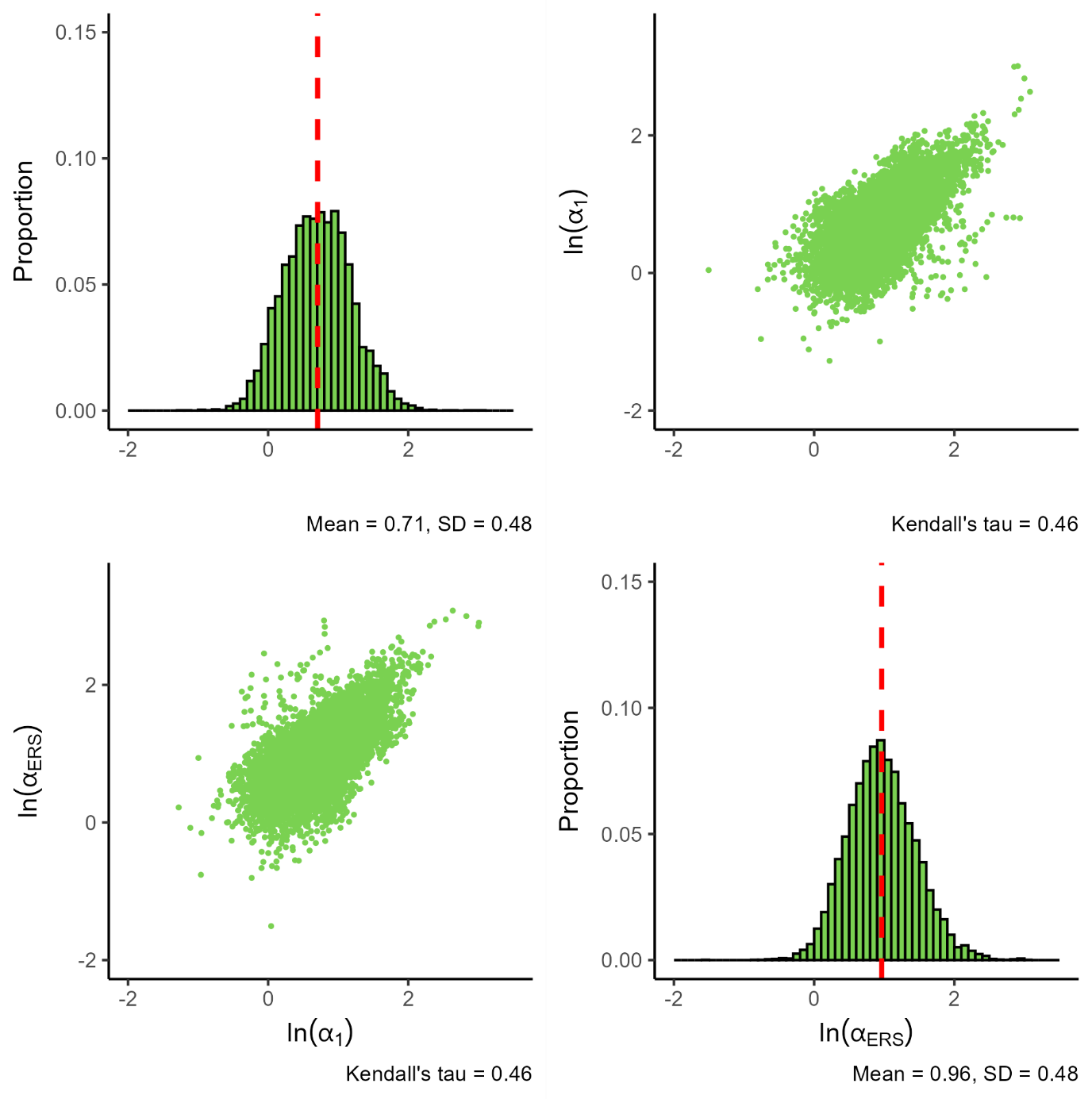
*Note.* $ln(\alpha_{1})$ denotes the natural logarithm of the substantive trait loading, and ${ln(\alpha}_{ERS})$ denotes the natural logarithm of the ERS loading. The red dashed line indicates the mean value of each parameter. All plots are based on 7837 item parameters from 1456 datasets, with each histogram bin being set to a width of 0.1.

Figure B2 contains the results for the IRTree $\alpha_{1}$. Note that the number of datasets and parameters is not identical across models, as some cases where the models did not converge had to be excluded for each model. Notably, the substantive trait loading of the IRTree appears much closer to the loading presented in the main paper than was the case for the MNRM (0.97 vs 0.94). This effects extends into the other MNRM and IRTree models considered in this paper. Several explanations for the fact that the MNRM substantive slope shifts substantially when no model selection is applied, while the IRTree slope remains relatively constant when no model selection is applied are possible. First, it may be that the large shift observed in the MNRM is the result of some kind of model misfit when applying the MNRM model to data where it is not preferred, while the IRTree does not suffer from this misfit when applied to data where it is not preferred (note that this reasoning could also be reversed: perhaps the relative absence of a shift for the IRTree is actually the result of IRTree misfit, while the MNRM shows us the “correct” shift). Second, it could be that the smaller change in the substantive trait loading for the IRTree actually has a similar impact to the MNRM’s larger change in substantive trait loading, and the effect merely looks small for the IRTree while being equally (or even more) impactful (we remind the reader that since the MNRM substantive trait loading concerns a polytomous slope and the IRTree substantive trait loading concerns a dichotomous item slope, their size cannot be directly compared). Finally, it may be that the IRTree merely shifts less since it was already preferred in more cases than the MNRM. To exclude this last possibility, we encourage the reader to view supplementary material C, where an explicit mean is calculated depending on which family of models is preferred. This analysis reveals that the third reason is not sufficient to explain the difference in substantive trait loading shifts between the families of models.

**Figure B2**

Parameters for the IRTree $\alpha_{1}$ regardless of model preference


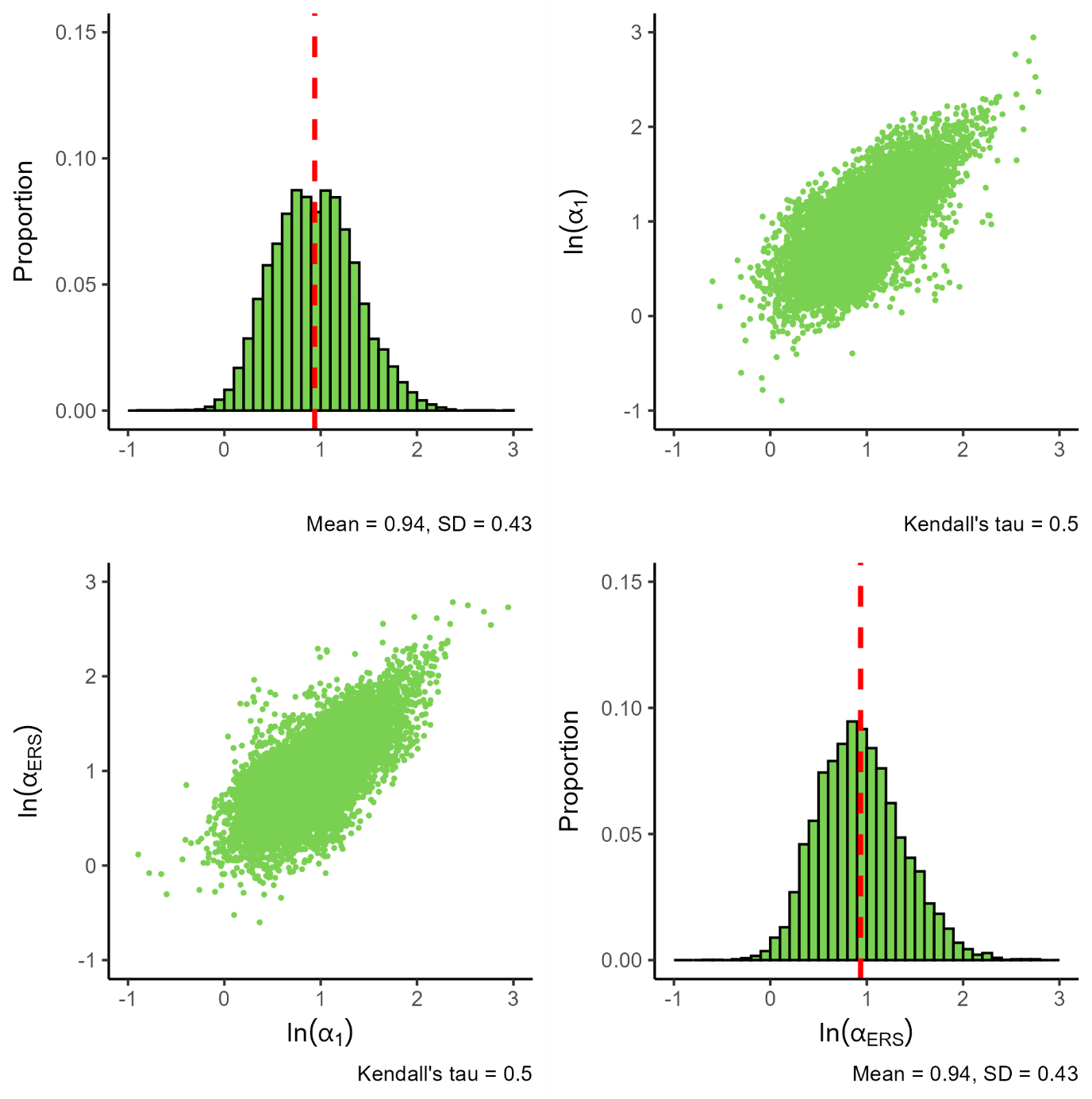


*Note.* $ln(\alpha_{1})$ denotes the natural logarithm of the substantive trait loading, and ${ln(\alpha}_{ERS})$ denotes the natural logarithm of the ERS loading. The red dashed line indicates the mean value of each parameter. All plots are based on 7842 item parameters from 1457 datasets, with each histogram bin being set to a width of 0.1.

Similar to the substantive trait loading, the mean log ERS loading hardly changes compared to the main paper (0.95 vs 0.94). The relationship between the substantive and ERS loading also does not change much (.52 vs .5).

Figures B3 and B4 present the results for the MNRM $b$. In Figure B2, we see that the mean substantive slope is again higher here than in the main paper (0.54 vs. 0.66). The mean log ERS slope is less affected (0.96 vs. 0.94), but the relationship between the two slopes increases slightly (.35 vs .42). Finally, the mean of the $b$ parameter in Figure B3 remains roughly the same (0.99 vs. 0.98). Notably, some more extreme $b$ values appear when we do not merely display cases where the model or a simpler version thereof is preferred. Four very extreme outliers (i.e., $b>4$) were excluded from all figures and mean calculations, since we cannot confidently conclude the model converged properly in these instances.

**Figure B3**

Parameters for the MNRM $b$ regardless of model preference


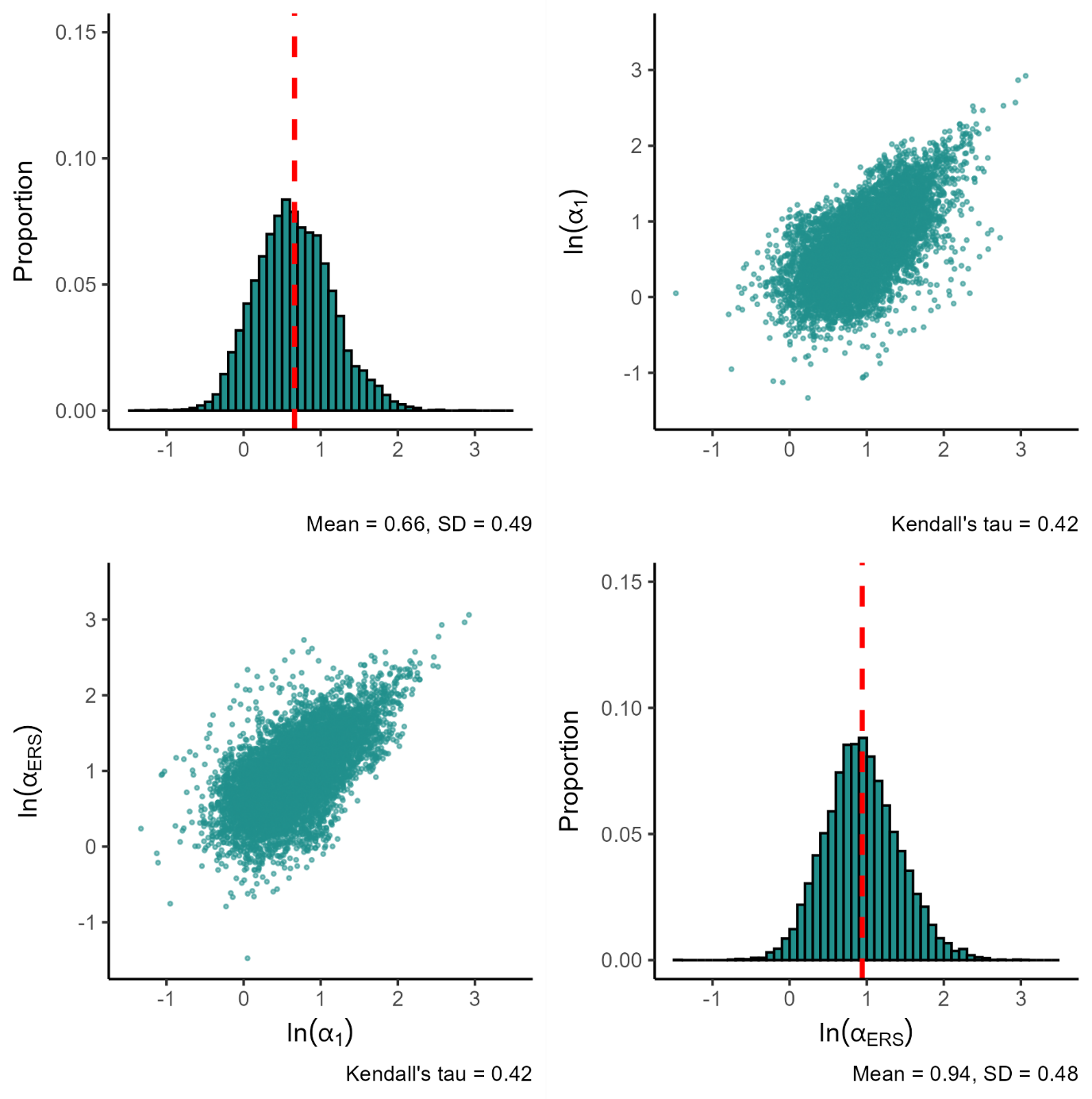


*Note.* $ln(\alpha_{1})$ denotes the natural logarithm of the substantive trait loading, and ${ln(\alpha}_{ERS})$ denotes the natural logarithm of the ERS loading. The red dashed line indicates the mean value of each parameter. All plots are based on 7733 item parameters from 1439 datasets, with each histogram bin being set to a width of 0.1.

**Figure B3**

$b$ parameter for the MNRM $b$ regardless of model preference


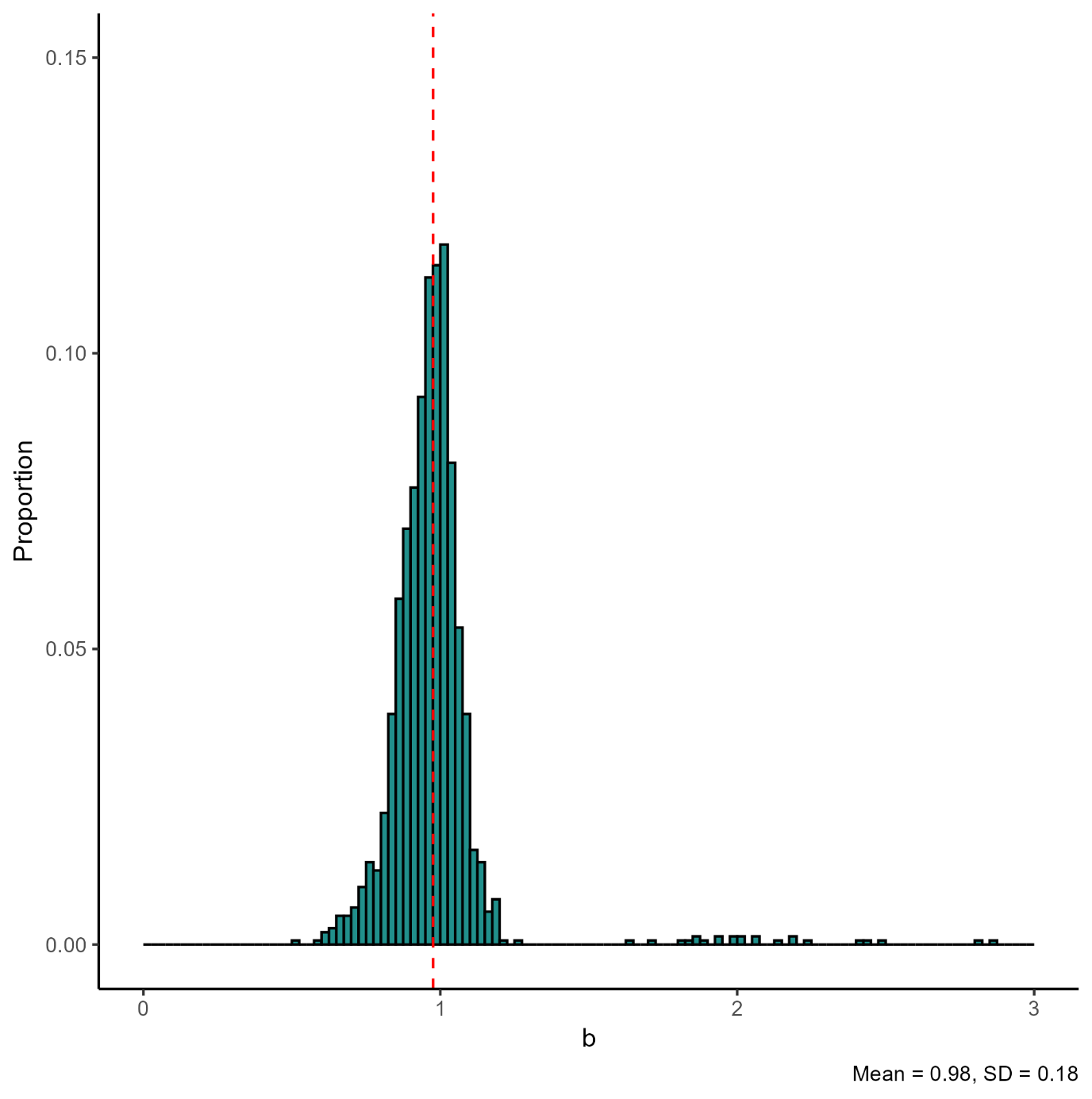


*Note.* The red dashed line indicates the mean value of the parameter. The plot is based on 1439 datasets, with each histogram bin being set to a width of 0.025.

Figures B4 and B5 contain the item parameters for the IRTree $P$. The mean of the log substantive slope (1 vs. 0.96) are not affected much, and the same holds for the mean log ERS slope (0.96 vs. 0.91), the relationship between the slopes (.46 vs. .43), and the mean $P$ value (0.96 vs. 0.95).

**Figure B4**

IRTree $P$ parameters regardless of model preference


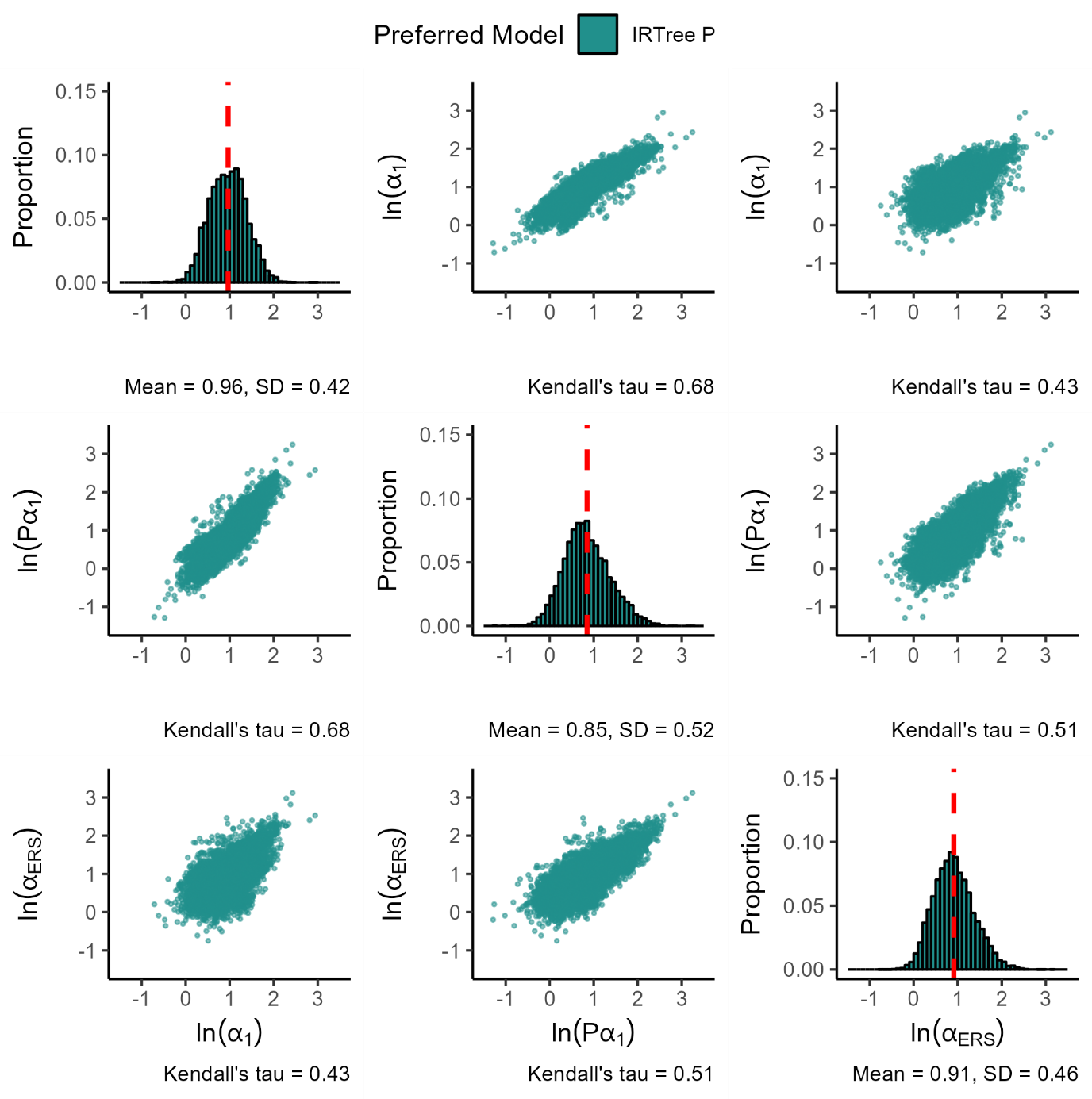


*Note.* $ln(\alpha_{1})$ denotes the natural logarithm of the substantive trait loading, and ${ln(\alpha}_{ERS})$ denotes the natural logarithm of the ERS loading. The red dashed line indicates the mean value of each parameter. All plots are based on 7829 item parameters from 1454 datasets, with each histogram bin being set to a width of 0.1.

**Figure B5**

$P$ parameter of the IRTree $P$ regardless of model preference


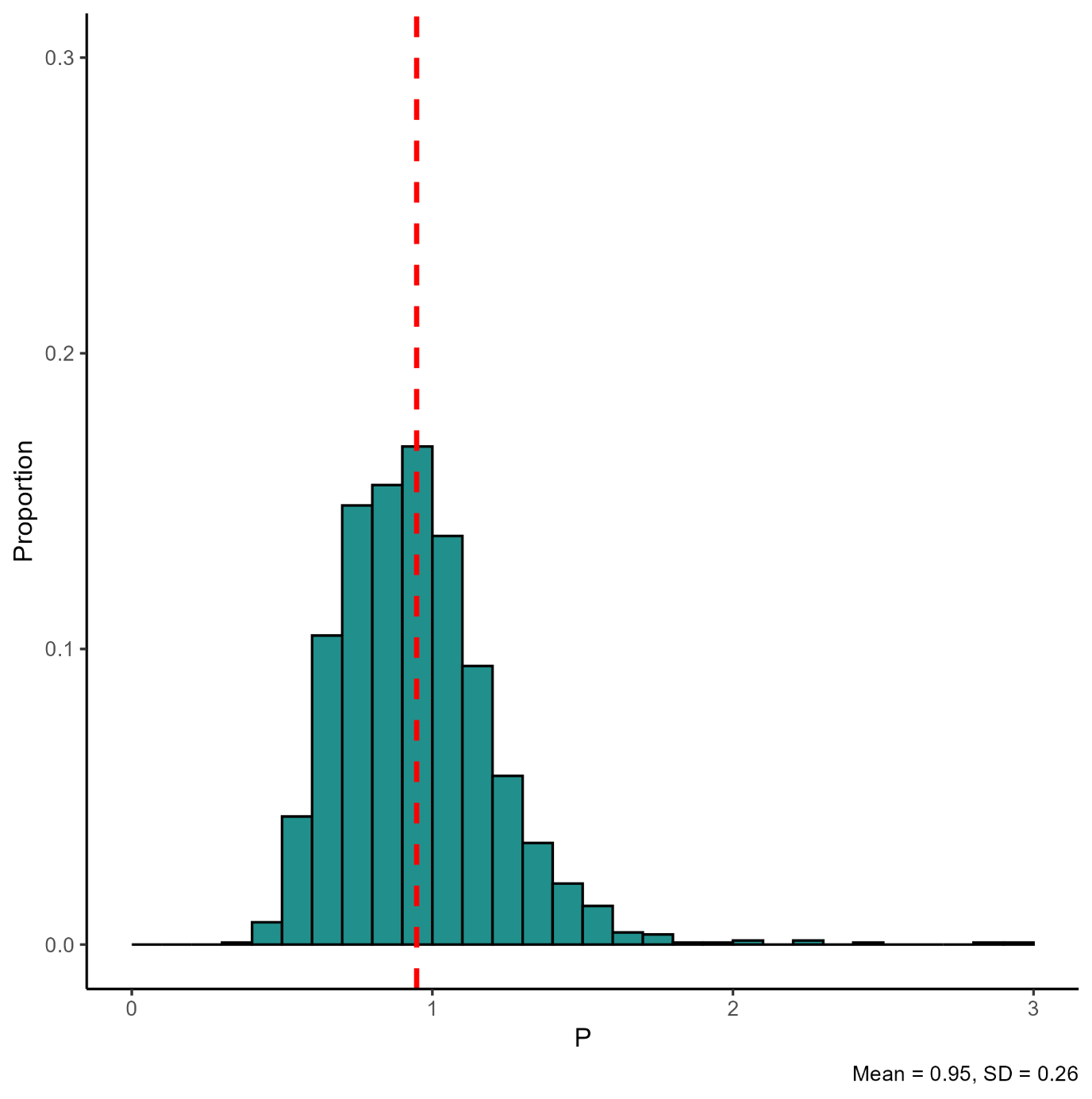


*Note.* The red dashed line indicates the mean value of the parameter. The plot is based on 1454 datasets, with each histogram bin being set to a width of 0.1.

Figure B6 contains the parameters for the MNRM $b_{i}$. The mean log substantive trait loading is again affected (0.57 vs. 0.66), with the mean log ERS loading (1 vs 0.95) and the mean $b$ parameter (0.93 vs. 0.96) being affected less. Overall, the relationship between the parameters is also similar (0.39 vs. 0.43 for the substantive loading, 0.18 vs. 0.21 for the ERS loading, and -0.01 vs. 0.07 for the $b$ parameter). Interestingly, the frequency of the $b$ values appears to decrease strongly after ~1.3, and then increase again somewhat around 2. This leads to two distinct clouds forming in each scatterplot involving the $b$ values.

**Figure B6**

MNRM $b_{i}$ parameters regardless of model preference


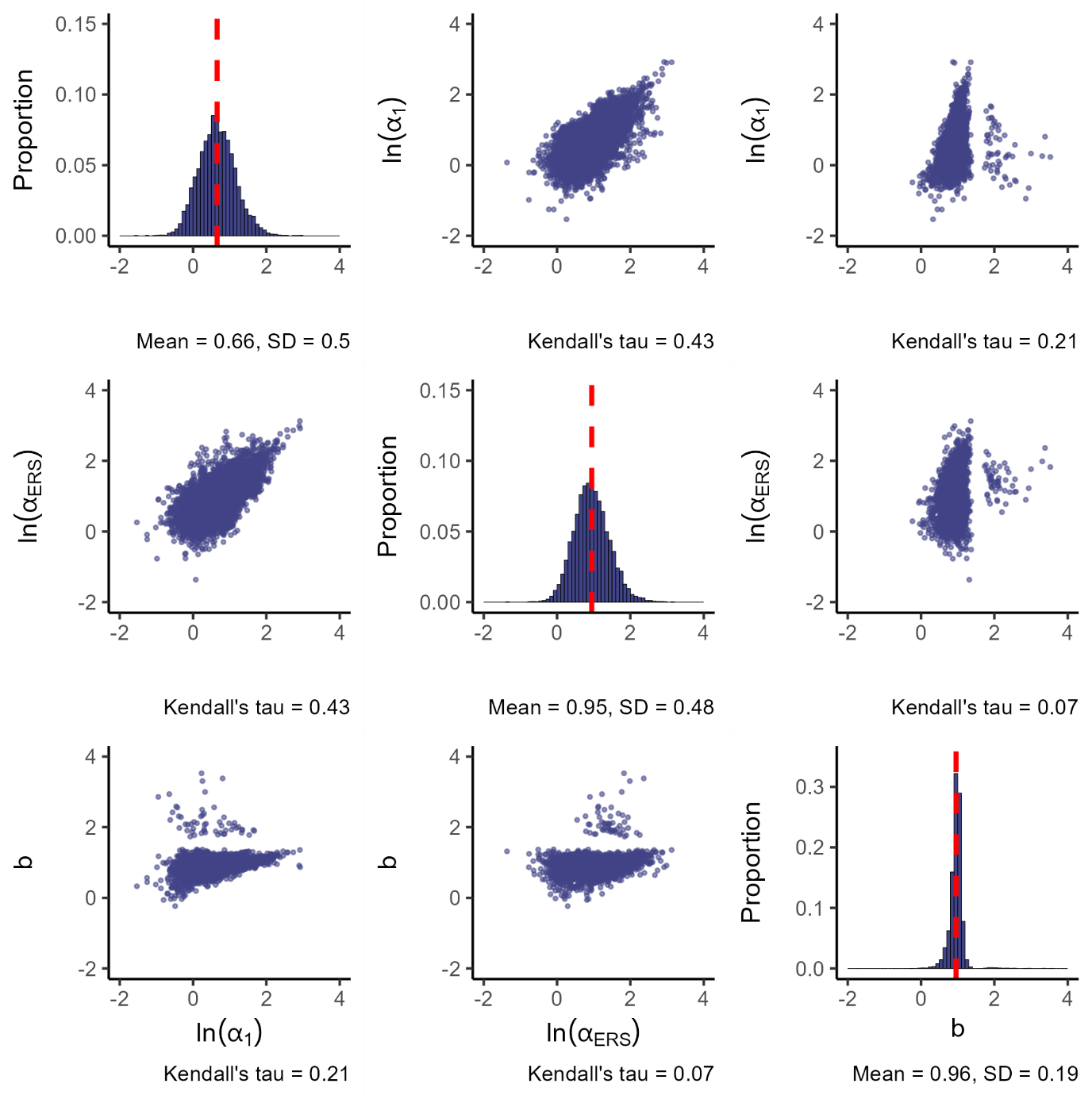


*Note.* $ln(\alpha_{1})$ denotes the natural logarithm of the substantive trait loading, and ${ln(\alpha}_{ERS})$ denotes the natural logarithm of the ERS loading. The red dashed line indicates the mean value of each parameter. All plots are based on 7723 item parameters from 1434 datasets, with each histogram bin being set to a width of 0.1.

Figure B7 contains the parameters for the IRTree model. Very little changes are visible for either the parameters (0.62 vs. 0.6 for the node 1 mean log substantive trait loading, 0.87 vs. 0.85 for the node 2 and 3 mean log substantive trait loading, and 0.92 vs. 0.92 for the mean log ERS loading) or the associations between them (0.62 vs. 0.6 for the node 1 and node 2/3 substantive trait, 0.43 vs. 0.42 for the node 1 substantive trait loading and ERS loading, and .57 vs. .54 for the node 2/3 and ERS loadings).

**Figure B7**

IRTree parameters regardless of model preference


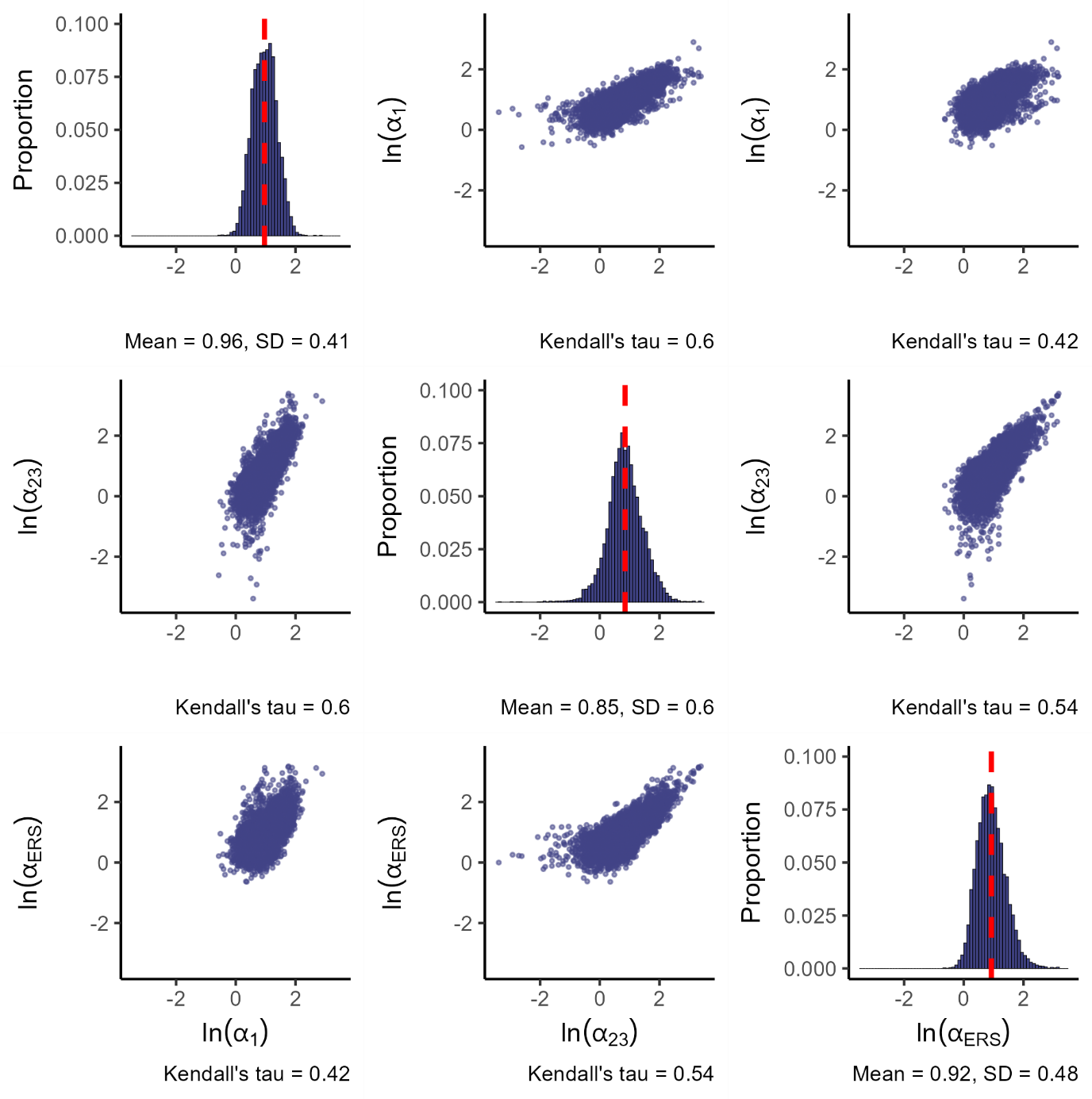


*Note.* $ln(\alpha_{1})$ denotes the natural logarithm of the substantive trait loading in node 1, $ln(\alpha_{23})$ denotes the natural logarithm of the substantive trait loading in nodes 2 and 3, ${ln(\alpha}_{ERS})$ denotes the natural logarithm of the ERS loading. The red dashed line indicates the mean value of each parameter. All plots are based on 7790 item parameters from 1447 datasets, with each histogram bin being set to a width of 0.1.

Finally, Figure B8 contains the correlations estimated by each model. We see that the correlation between the substantive trait and the ERS trait is now not on average substantially negative for the MNRM models as it was in the main paper. Instead, all models obtain a mean correlation around zero. While we could not validly compare the substantive trait loadings from the different models to each other, we can compare the correlations produced by the models, and do so in Figure C4. Findings from this Figure make the explanation of model misfit resulting in biased parameters somewhat unlikely for the correlations. We thus conclude the information criteria genuinely tend to prefer the MNRM in cases where the correlation between ERS and the substantive trait is somewhat lower on average.

**Figure B8**

Correlations between the substantive trait and ERS for all models regardless of model preference


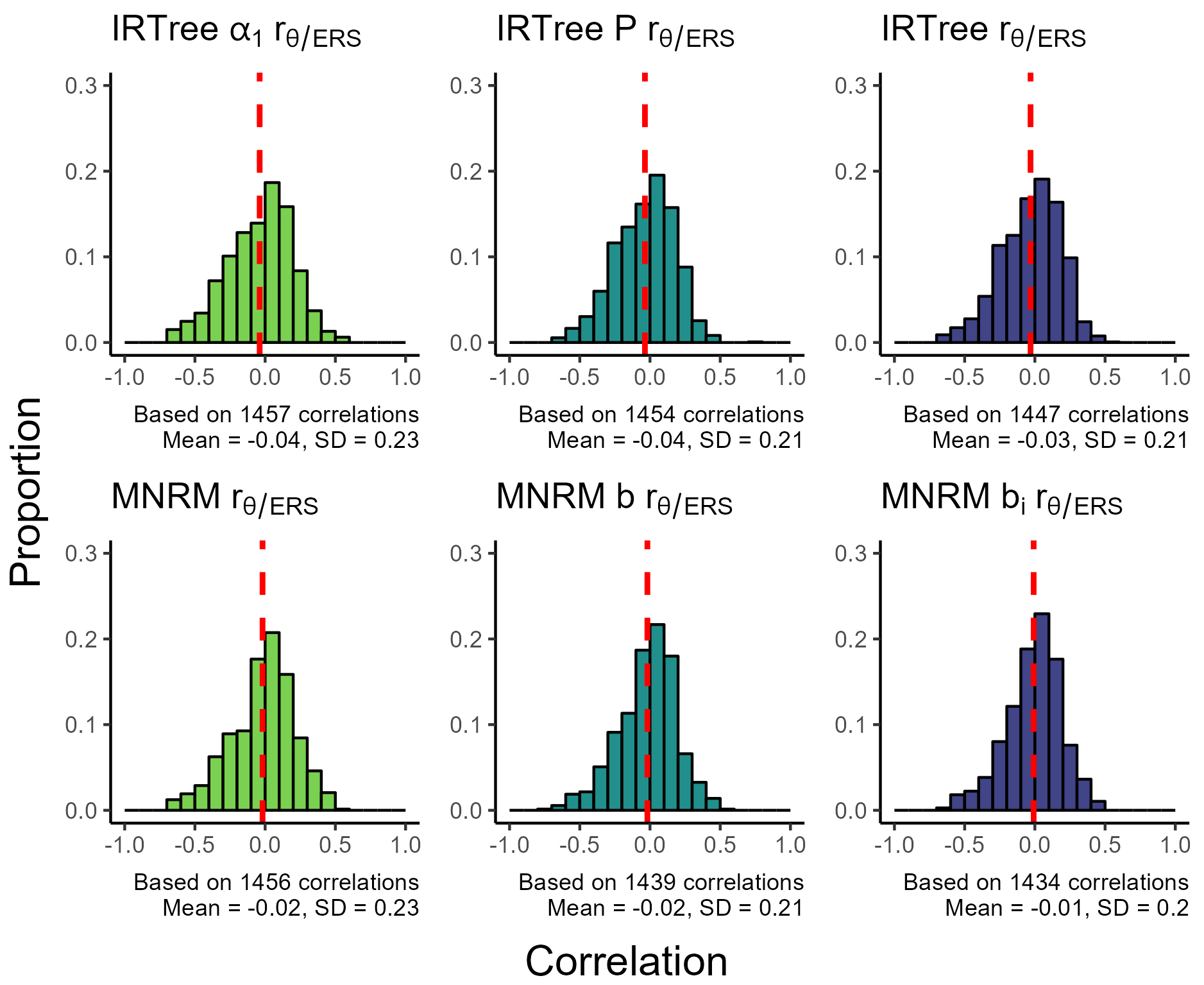


*Note.* $r_{\theta/ERS}$ denotes the correlation between the substantive trait and ERS. The red dashed line indicates the mean value of each parameter. Each histogram bin was set to a width of 0.1. Again note the number of correlations per model differs because not all models converged in every dataset.

**Supplementary Material C**

**MNRM** $\boldsymbol{b}_{\boldsymbol{i}}$ **and IRTree parameters split depending on which family of models was preferred**

In this supplementary material, we investigate the selection effects mentioned in the main paper and supplementary material B further. Specifically, we wished to gain a more precise overview of the differences between the mean estimated parameters depending on which family of models was preferred. To achieve this, we fit the most complex models of each family (MNRM $b_{i}$ and IRTree) to every dataset. Note that we chose to fit the most complex models of each family to avoid “within-family” misfit, and instead ensure all misfit came from fitting an IRTree rather than MNRM model or the reverse. Then, we split the data into cases where an IRTree family model was preferred and cases where an MRNM model was preferred, and plotted the distribution and calculated the mean for each.

Figure C1 contains the outcome of this process for the MNRM $b_{i}$. As we can see, the estimated substantive trait loading is far lower in cases where the MNRM family of models is preferred than in cases where the IRTree family of models is preferred (0.58 vs. 0.93). Assuming we can trust the estimated value of the MNRM $b_{i}$ in cases where an IRTree model was preferred, this could indicate MNRM models are more likely to be selected by information criteria in datasets with a weak substantive trait loading. For the ERS (1 vs 0.93) and $b$ parameters (0.93 vs. 0.96), this effect is much less pronounced.

**Figure C1**

MNRM $b_{i}$ parameters split between cases where the IRTree family of models is preferred versus cases where the MNRM family of models is preferred.


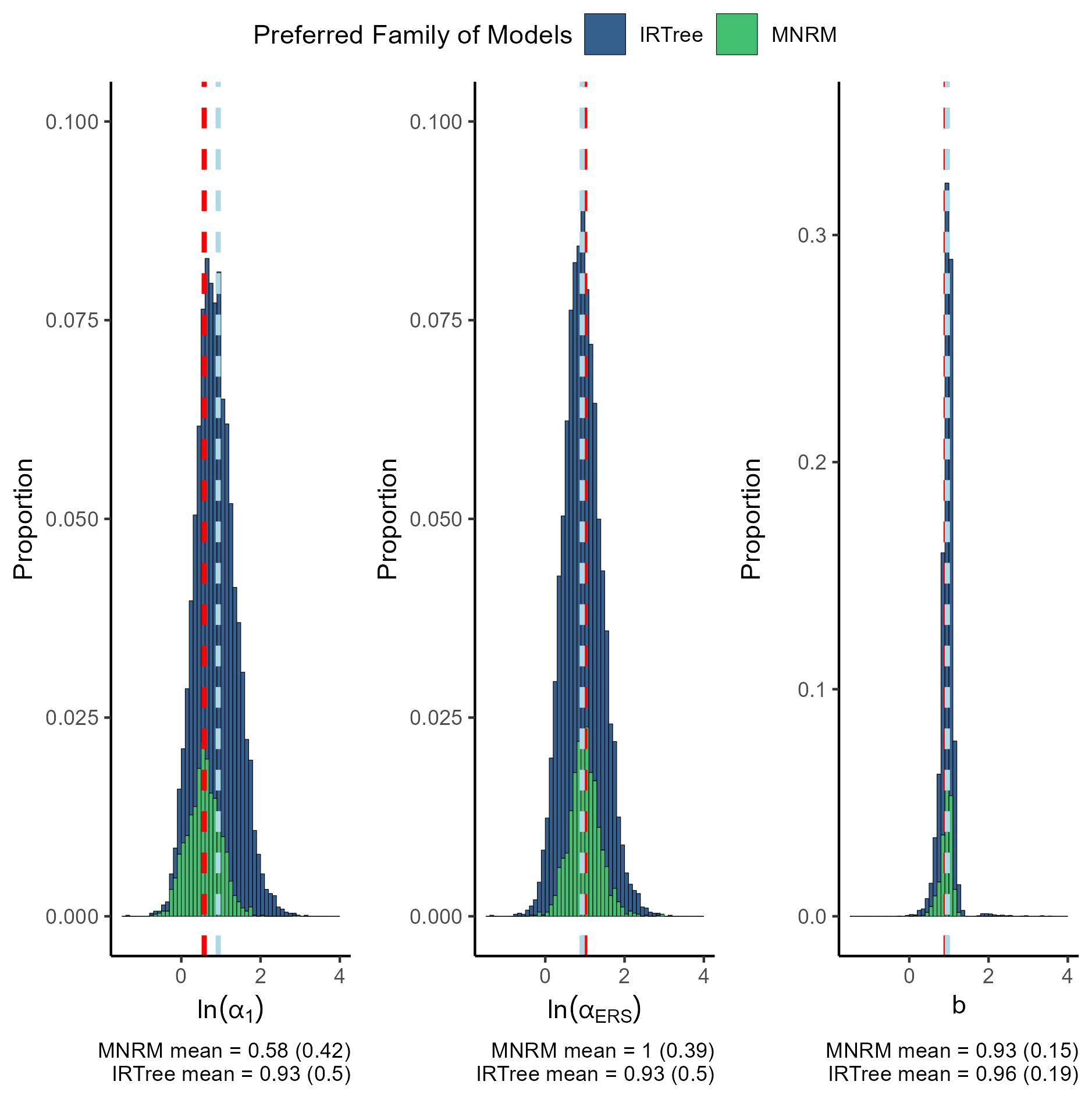


*Note.* $ln(\alpha_{1})$ denotes the natural logarithm of the substantive trait loading, and ${ln(\alpha}_{ERS})$ denotes the natural logarithm of the ERS loading. The red dashed line indicates the mean value of each parameter when a model from the MNRM family was preferred, with the light blue line indication the mean value of each parameter when a model from the IRTree family was preferred. All plots are based on 7684 item parameters from 1434 datasets, with each histogram bin being set to a width of 0.1 and bars stacked on top of each other. Note that the model contains less parameters than in the main paper, since only cases where both an IRTree and MNRM $b_{i}$ model converged were included.

Figure C2 is similar to plot C1, but now contains the parameters of the IRTree model. Some differences are again visible. First, the mean substantive trait loading in node 1 is somewhat lower in cases where an MRNM family model was preferred (0.9 vs. 0.97). Second, the mean substantive trait loading in nodes 2 and 3 was quite a bit lower in cases where an MRNM family model was preferred (0.73 vs. 0.87). While the decrease in mean log substantive trait slopes is thus visible for the IRTree in all nodes when an MNRM model is preferred, this decrease is substantially smaller for the IRTree than for the MNRM $b_{i}$. The possible explanations for this asymmetry between the changes in substantive trait loading parameters for the different models are similar to those presented in supplementary material B (The IRTree may be affected by misfit differently when applied to an MNRM dataset than the MNRM $b_{i}$ is when applied to an IRTree dataset, or the relatively smaller shift in the IRTree may actually be equally or more impactful than the shift in the MNRM $b_{i}$). Findings here do however show it is not the case that the shift for the IRTree is merely smaller because it was already preferred in more cases. Finally, the ERS loading was relatively unaffected by which family of models was preferred (0.94 vs. 0.91).

**Figure C2**

IRTree parameters split between cases where the IRTree family of models is preferred versus cases where the MNRM family of models is preferred.


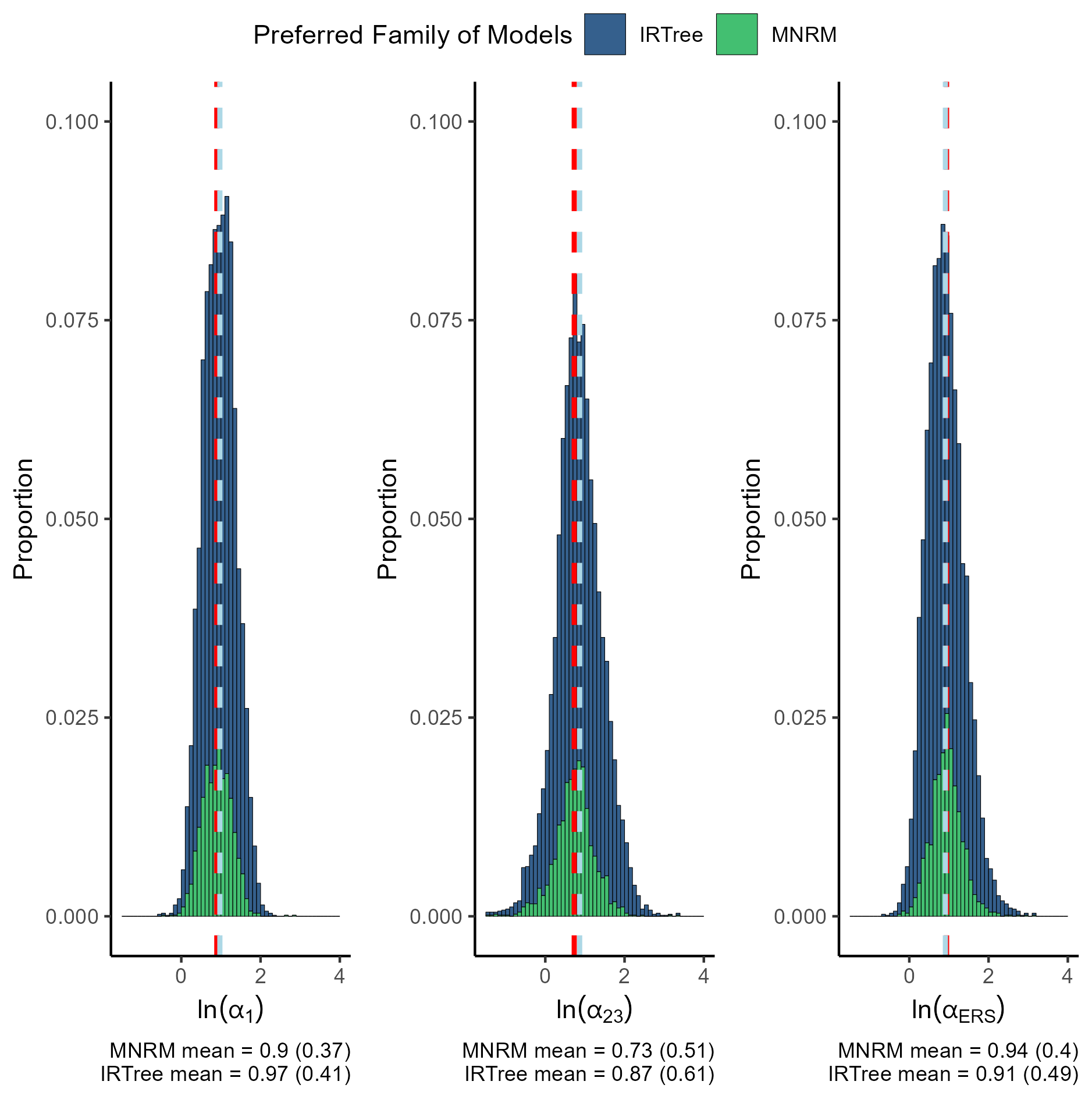


*Note.* $ln(\alpha_{1})$ denotes the natural logarithm of the substantive trait loading in node 1, $ln(\alpha_{23})$ denotes the natural logarithm of the node 2/3 substantive loading, and ${ln(\alpha}_{ERS})$ denotes the natural logarithm of the ERS loading. The red dashed line indicates the mean value of each parameter when a model from the MNRM family was preferred, with the light blue line indication the mean value of each parameter when a model from the IRTree family was preferred. All plots are based on 7684 item parameters, with each histogram bin being set to a width of 0.1 and bars stacked on top of each other.

Figure C3 displays the estimated correlations for both the MNRM $b_{i}$ and the IRTree model split depending on which model family was preferred. In the Figure, we see that for both the MRNM and the IRTree model, the correlation was estimated to be lower in cases where an MNRM model was preferred than in cases where an IRTree model was preferred (-.14 vs .02 for the MNRM $b_{i}$, and -.15 vs. 0 for the IRTree). This may point to the fact that MNRM models tend to be preferred in cases where the correlation between ERS and the substantive trait is negative.

**Figure C3**

Estimated correlations between ERS and the substantive trait from the MNRM $b_{i}$ (left) and the IRTree (right) split by which family of models was preferred.


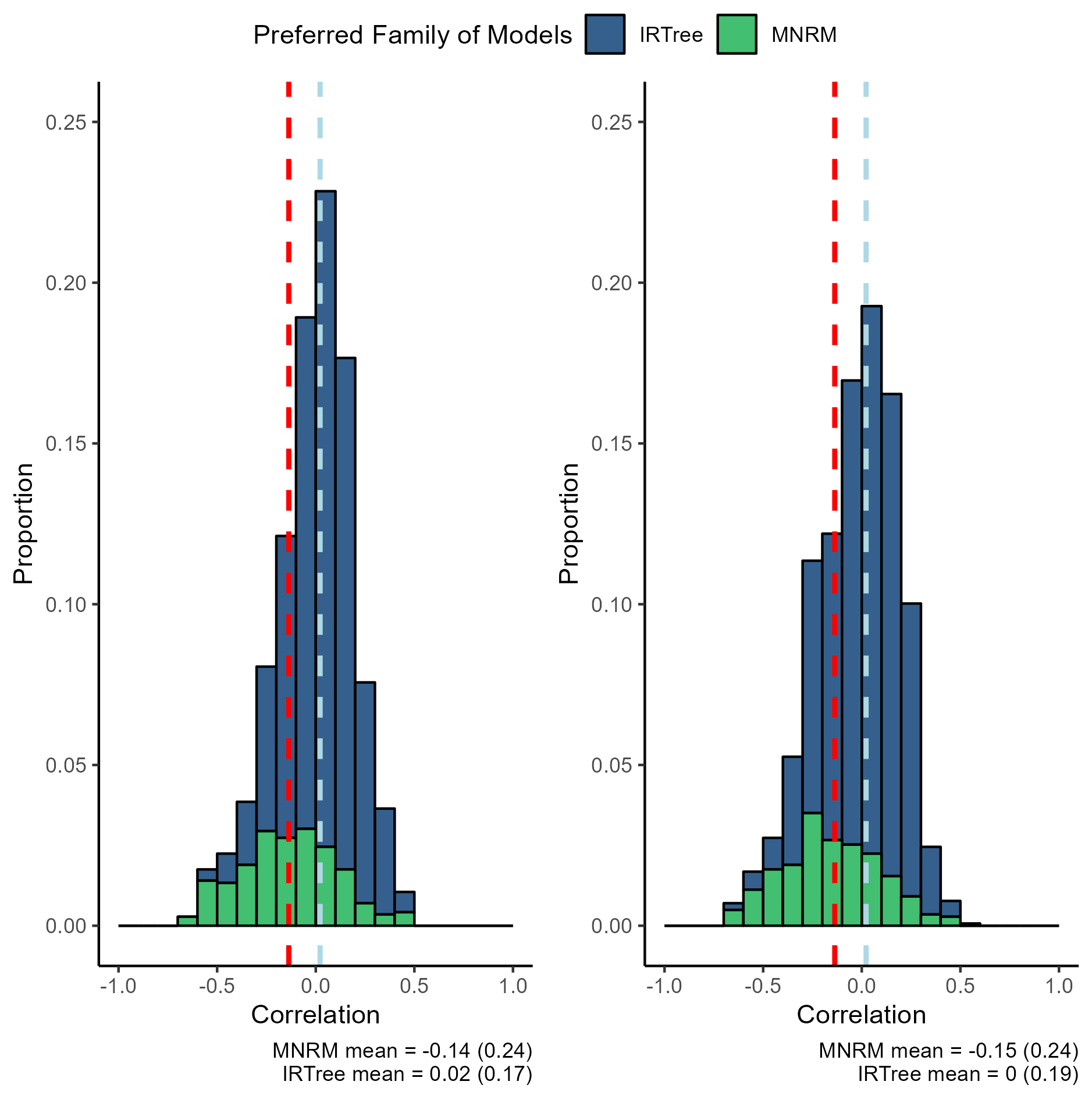


*Note.* The red dashed line indicates the mean value of each correlation when a model from the MNRM family was preferred, with the light blue line indication the mean value of each correlation when a model from the IRTree family was preferred. Both plots are based on 1427 datasets, with each histogram bin being set to a width of 0.1 and bars stacked on top of each other.

To further exclude the possibility that the difference between the correlations when different families of models were preferred was caused by some kind of model misfit, we compared the estimates for the correlation between the ERS and substantive trait in Figure C4. In the Figure, we see that estimates tend to be similar, although some (~15) cases of notable disagreement appear. The general agreement on the magnitude of the correlation between ERS and the substantive trait regardless of which family of models was preferred and which model was applied to the data points to the fact there is a genuine selection effect. In other words, the MNRM tends to be preferred in cases where the correlation between ERS and the substantive trait is on average lower.

**Figure C4**

Scatterplot of estimated correlations from the IRTree and MNRM $b_{i}$ models


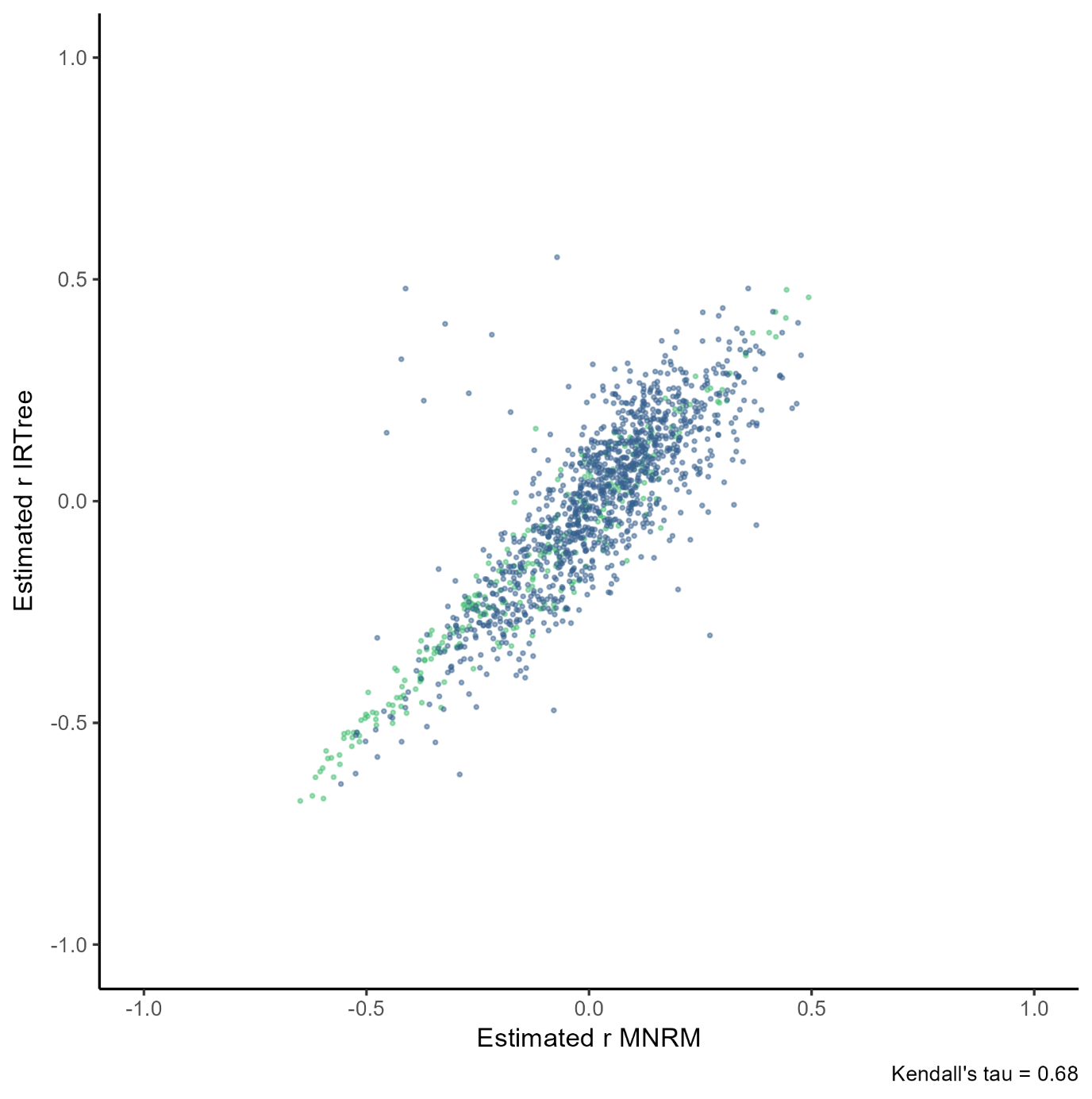


*Note.* This plot is based on 1427 datasets, with each dot representing an estimated correlation for the IRTree and MNRM $b_{i}$ model. Green dots indicate a case where an MNRM family model was preferred, and blue dots indicate a case where an IRTree family model was preferred.
